# Study of *Mycobacterium tuberculosis* Complex Genotypic Diversity in Malaysia Reveals a Predominance of Ancestral East-African-Indian Lineage with a Malaysia-Specific Signature

**DOI:** 10.1371/journal.pone.0114832

**Published:** 2014-12-11

**Authors:** Fazli Ismail, David Couvin, Izzah Farakhin, Zaidah Abdul Rahman, Nalin Rastogi, Siti Suraiya

**Affiliations:** 1 Department of Medical Microbiology & Parasitology, School of Medical Sciences, Universiti Sains Malaysia, Kota Bharu, Kelantan, Malaysia; 2 WHO Supranational TB Reference Laboratory, Institut Pasteur de la Guadeloupe, Abymes, Guadeloupe, France; University of Minnesota, United States of America

## Abstract

**Background:**

Tuberculosis (TB) still constitutes a major public health problem in Malaysia. The identification and genotyping based characterization of *Mycobacterium tuberculosis* complex (MTBC) isolates causing the disease is important to determine the effectiveness of the control and surveillance programs.

**Objectives:**

This study intended a first assessment of spoligotyping-based MTBC genotypic diversity in Malaysia followed by a comparison of strains with those prevailing in neighboring countries by comparison with an international MTBC genotyping database.

**Methods:**

Spoligotyping was performed on a total of 220 *M. tuberculosis* clinical isolates collected in Kelantan and Kuala Lumpur. The results were compared with the SITVIT2 international database of the Pasteur Institute of Guadeloupe.

**Results:**

Spoligotyping revealed 77 different patterns: 22 corresponded to orphan patterns while 55 patterns containing 198 isolates were assigned a Spoligo International Type (SIT) designation in the database (the latter included 6 newly created SITs). The eight most common SITs grouped 141 isolates (5 to 56 strains per cluster) as follows: SIT1/Beijing, n = 56, 25.5%; SIT745/EAI1-SOM, n = 33, 15.0%; SIT591/EAI6-BGD1, n = 13, 5.9%; SIT256/EAI5, n = 12, 5.5%; SIT236/EAI5, n = 10, 4.6%; SIT19/EAI2-Manila, n = 9, 4.1%; SIT89/EAI2-Nonthaburi, n = 5, 2.3%; and SIT50/H3, n = 3, 1.4%. The association between city of isolation and lineages was statistically significant; Haarlem and T lineages being higher in Kuala Lumpur (p<0.01). However, no statistically significant differences were noted when comparing drug resistance vs. major lineages, nor between gender and clades.

**Conclusions:**

The ancestral East-African-Indian (EAI) lineage was most predominant followed by the Beijing lineage. A comparison of strains with those prevailing in neighboring countries in South Asia, East Asia and South East Asia underlined the phylogeographical specificity of SIT745 for Malaysia, and its probable ongoing evolution with locally evolved strains sharing a specific signature characterized by absence of spacers 37, 38, and 40. Pending complementary genotyping confirmation, we propose that SIT745/EAI-SOM is tentatively reclassified as SIT745/EAI-MYS.

## Introduction

Despite a recently observed decrease in number of Tuberculosis (TB) cases, in 2012 it led to an estimated 8.6 million new cases and 1.3 million death worldwide – including 320,000 deaths among TB/HIV co infected patients [1]. Furthermore, the present rate of decline (2.2% per year) is insufficient to reach the target for the elimination of TB by 2050 in the world, defined as ≤1 TB case per million population per year [1]. With regards to Malaysia, with more than 19,337 cases reported in 2010, TB was notified as the second highest infectious disease after dengue fever in 2010, and remains a major public health challenge [2]. It is obvious that a better understanding of local TB transmission would benefit both the infection control and management, and would ultimately lead to better patient care. This goal would require a better understanding of how tubercle bacilli are transmitted, which clones are involved in ongoing transmission and drug resistance, and identify subpopulations at risk – questions that would prove difficult to answer without the support of molecular epidemiology.

An overview of molecular epidemiology of *M. tuberculosis* strains circulating in Malaysia was reported by Dale *et al.* in 1999 [3]. In the study, 439 isolates were genotyped using IS*6110*-Restriction Fragment Length Polymorphism (RFLP) and strains were compared between Peninsular Malaysia and East Malaysia. However, there was limited information reported on strain association with patients' demographic data and the phylogenetic lineages involved. Since recent studies have reported that certain *M. tuberculosis* lineages may be associated with particular clinical phenotypes and epidemiologic features [4]–[6], we decided to undertake a first assessment of MTBC genotypic diversity. Based on recent reviews on *Mycobacterium tuberculosis* complex (MTBC) molecular typing methods [7] and strategies and innovations in the broad field of TB molecular epidemiology [8], we opted for spoligotyping which is one of the most widely used methods for an initial assessment of MTBC genotypic diversity. This polymerase chain reaction (PCR)-based technique relies on the presence or absence of 43 unique spacer sequences in the direct repeat (DR) locus of the *M. tuberculosis* genome, useful both for simultaneous detection and typing of *M. tuberculosis* strains [9]. This methodology is ideal for assigning MTBC strains in major phylogenetic lineages thanks to the SITVIT2 international database, which further allows one to compare strains worldwide and draw distribution maps [10]. We selected MTBC isolates from TB patients residing in 2 different geographic locations (Kelantan and Kuala Lumpur), and looked for significant associations between phylogenetic lineages vs. demographic and epidemiologic characteristics, in addition to evolutionary relationships between circulating MTBC isolates in Malaysia.

## Materials and Methods

### Patient population and clinical isolates

*M. tuberculosis* isolates were collected from tertiary hospitals in Kelantan (n = 184) and Kuala Lumpur (n = 36), Malaysia between 2009 and 2013 (1 isolate per patient). Diagnostic specimens were cultured and isolated on Ogawa media after decontamination. Drug Susceptibility testing (DST) against first-line anti-TB drugs (rifampicin, isoniazid, streptomycin, and ethambutol) was done by The National Public Health Laboratory (NPHL) using the proportional method [11]. Clinical and demographic data (date of isolation, isolation city, age, sex, ethnicity, first line-drug susceptibility patterns) were retrieved from TBIS 101A registration book and Laboratory Information System (LIS).

### DNA extraction

Mycobacterial DNA was extracted from all *M. tuberculosis* by a simple boiling method. Briefly, a loopful of bacteria was suspended in 200 µl of distilled water, vortexed and briefly centrifuged. The cells were heat-killed for 10 minutes at 100°C and immediately put on ice for 15 minutes. The bacterial suspensions were centrifuged at 13,000 rpm for 5 minutes. The supernatants were transferred to a new microcentrifuge tube and used in PCR analysis or stored at −20°C.

### Spoligotyping and database comparison

Spoligotyping was carried out according to the protocol previously described by Kamerbeek *et al* [9], using a commercially available kit (Ocimum Biosolutions Inc., Hyderabad, India). *M. tuberculosis* H37Rv and *M. bovis* BCG DNAs were used as parallel positive controls and distilled water as a negative control. The patterns obtained were compared by using the SITVIT2 proprietary database of the Pasteur Institute of Guadeloupe, which is an updated in-house version of the publicly released SITVITWEB database [10]; available online at http://www.pasteur-guadeloupe.fr:8081/SITVIT_ONLINE/]. In this database, Spoligotype International Type (SIT) designates an identical pattern shared by two or more patient isolates, whereas “orphan” designates patterns reported for a single isolate that does not correspond to any of the strains recorded in the database repository.

### Phylogenetical analysis

Major phylogenetic clades were assigned according to signatures provided in SITVITWEB [10] for definition of variants within 62 existing lineages/sub-lineages. These include specific signatures for various MTBC members, as well as rules defining major lineages/sub-lineages for *M. tuberculosis* stricto sensu, i.e., the Beijing clade, the Central Asian (CAS) clade and two sublineages, the East African-Indian (EAI) clade and nine sublineages, the Haarlem (H) clade and three sublineages, the Latin American-Mediterranean (LAM) clade and 12 sublineages, the “Manu” family and three sublineages, the S clade, the IS*6110* – low-banding × clade and four sublineages, and an ill-defined T clade with five sublineages. Note that with their definitive reclassification pending, we refer to LAM10-CAM (prototype SIT61) as the Cameroon lineage, LAM7-TUR as the Turkey lineage, and some spoligotypes previously classified among H3/H4 sublineages as H3/Ural-1 and H4/Ural-2 [12]; the latter include patterns belonging to H4 sublineage that were relabeled "Ural-2", and some patterns previously classified as H3 sublineage but with an additional specific signature (presence of spacer 2, absence of spacers 29 to 31, and 33 to 36), that are now relabeled "Ural-1" [13].

The BioNumerics software Version 6.6 (Applied Maths NV, Sint-Martens-Latem, Belgium) was used to build minimum spanning trees (MST) based on spoligotyping data. MST is an undirected network in which all of the isolates are linked together with the fewest possible linkages between nearest neighbors, represented by branches (continuous vs. dashed and dotted lines). Note that the length of the branches represents the distance between patterns while the complexity of the lines (continuous, gray dashed and gray dotted) denotes the number of allele/spacer changes between two patterns: solid lines, 1 or 2 or 3 changes (thicker ones indicate a single change, while the thinner ones indicate 2 or 3 changes); gray dashed lines represent 4 changes; and gray dotted lines represent 5 or more changes.

Spoligoforest tree based on Fruchterman-Reingold algorithm was drawn using the SpolTools software [14], [15]. Contrary to the MST, the Spoligoforest trees are directed graphs which only evolve by loss of spacers, represented by directed edges between nodes, with the arrowheads pointing to descendant spoligotypes. In this representation, the heuristic approach used selects a single inbound edge with a maximum weight using a Zipf model. Solid black lines link patterns that are very similar, i.e., loss of one spacer only (maximum weight being 1.0), while dashed lines represent links of weight comprised between 0.5 and 1, and dotted lines a weight less than 0.5. In a Spoligoforest, nodes are not necessarily all connected (indeed, in case of too many changes between two strains, there are no edges linking them. GraphViz software (http://www.graphviz.org) was used to color the strains based on their lineages on the Spoligoforest trees [16].

### Worldwide distribution analysis

The predominant MTBC SITs/lineages obtained in our study (SITs containing >1% i.e., 3 or more isolates) were screened for their worldwide distribution analysis using the SITVIT2 database country-wise as well as macro-geographically as United Nations sub-regions (http://unstats.un.org/unsd/methods/m49/m49regin.htm): AFRI (Africa), AMER (Americas), ASIA (Asia), EURO (Europe), and OCE (Oceania), subdivided in: E (Eastern), M (Middle), C (Central), N (Northern), S (Southern), SE (South-Eastern), and W (Western). In this classification scheme, CARIB (Caribbean) belongs to Americas, while Oceania is subdivided in 4 sub-regions, AUST (Australasia), MEL (Melanesia), MIC (Micronesia), and POLY (Polynesia). Note that Russia was attributed a new sub-region by itself (Northern Asia) instead of including it among the rest of Eastern Europe.

### Mapping in neighboring countries

We also compared MTBC population structure in our study vs. results retrieved from the SITVIT2 database for neighboring countries in South Asia n = 4271 strains (India n = 2647, Bangladesh n = 1090, Pakistan n = 385, Nepal n = 49, Sri-Lanka n = 100), East Asia n = 5216 strains (China n = 3072, Japan n = 1569, South Korea n = 152, Taiwan n = 423), and South East Asia n = 3059 strains (Indonesia n = 422, Myanmar n = 310, Malaysia without our present study n = 749, Philippines n = 155, Singapore n = 333, Thailand n = 307, Vietnam n = 783).

### Statistical analysis

Statistical analyses were performed using the Statistical Package for the Social Sciences (SPSS) version 18.0 and STATA version 10.1. Associations between *M. tuberculosis* clades with patient demographic and drug resistant criteria were assessed using Chi-square analysis and Fisher's exact test. P-values of ≤0.05 were considered statistically significant and the 95% confidence interval (95% CI) was calculated. Mean and standard deviation (SD) were calculated for age of the patients.

### Ethics statement

This retrospective genotyping study was approved by The Human Research Ethics Committee, Universiti Sains Malaysia (FWA Reg. No: 00007718; IRB Reg. No: 00004494) on 27th March 2013. Because the retrospective review of the TB registry and medical records, inform consents were not available. All patients' record and information was anonymized and de-identified prior to analysis.

## Results

Altogether, 83.6% of *M. tuberculosis* isolates were from patients in Kelantan and 16.4% were from Kuala Lumpur. Regarding the 3 prevailing ethnic groups in 2010 Malaysian census (60.3% Malays, 22.9% Chinese, and 7.1% Indians; http://en.wikipedia.org/wiki/Demographics_of_Malaysia), the proportion of TB patients in our study was 93.2% for Malays, 5.0% for the Chinese, and 1.8% for Indians. The mean age of the patients was 49.2 years (SD 17.9 years, range 6–89 years). Almost two third of patients (62.7%) were male with a male to female sex-ratio of 1.68. Drug susceptibility patterns available for 143 (65.0%) of isolates showed that 125 (87.4%) were pansusceptible while 18 were drug-resistant (including a single multidrug-resistant isolate). However, the drug susceptibility results of 77 (35.0%) of isolates were not available due to several factors including data loss and contamination of the culture during the process.

Spoligotyping of the study sample (n = 220 isolates) as well as the detailed results obtained including demographic, epidemiologic, drug-resistance, and genotyping information are summarized in **S1 Table**. Spoligotyping revealed 77 different patterns: 22 corresponded to orphan patterns not yet reported in the SITVIT2 database, while 55 patterns containing 198 isolates were assigned a Spoligo International Type (SIT) designation in the database (the latter included 6 “newly created” SITs containing 11 isolates from this study). Refer to **S1 Table** for a listing of orphan patterns (highlighted in blue), and **Table 1** for a description of 55 SITs. Note that SITs followed by an asterisk indicate "newly created” SITs due to 2 or more strains belonging to an identical new pattern within this study or after a match with an orphan in the database (SITs followed by number of strains: SIT3993* this study n = 1, MYS n = 1; SIT3994* this study n = 2, MYS n = 1; SIT3994* this study n = 2; SIT3995* this study n = 2; SIT3996* this study n = 2; SIT3997* this study n = 2; 3998* this study n = 2. Note that the 3 letter country codes are according to http://en.wikipedia.org/wiki/ISO_3166-1_alpha-3).

**Table 1:** Description of 55 shared-types (SITs; n = 198 isolates) and corresponding spoligotyping defined lineages/sublineages starting from a total of 220 *M. tuberculosis* strains isolated in Malaysia.

SIT a	Spoligotype Description	Octal Number	Nb (%) in study	% in study vs. Database	Clade b	Unique or Clustered SIT c
1	□□□□□□□□□□□□□□□□□□□□□□□□□□□□□□□□□□▪▪▪▪▪▪▪▪▪	000000000003771	56 (25.45)	0.54	Beijing	Clustered
11	▪□□▪▪▪▪▪▪▪▪▪▪▪▪▪▪▪▪▪▪▪▪▪▪▪▪▪□□□□▪□▪▪□□□▪▪▪▪	477777777413071	1 (0.45)	0.15	EAI3-IND	Unique
19	▪▪□▪▪▪▪▪▪▪▪▪▪▪▪▪▪▪▪□□▪▪▪▪▪▪▪□□□□▪□▪▪▪▪▪▪▪▪▪	677777477413771	9 (4.09)	0.99	EAI2-Manila	Clustered
26	▪▪▪□□□□▪▪▪▪▪▪▪▪▪▪▪▪▪▪▪□□□□□□□□□□□□▪▪▪▪▪▪▪▪▪	703777740003771	1 (0.45)	0.06	CAS1-Delhi	Unique
34	▪▪▪▪▪▪▪▪□□▪▪▪▪▪▪▪▪▪▪▪▪▪▪▪▪▪▪▪▪▪▪□□□□▪▪▪▪▪▪▪	776377777760771	1 (0.45)	0.12	S	Unique
48	▪▪▪▪▪▪▪▪▪▪▪▪▪▪▪▪▪▪▪▪▪▪▪▪▪▪▪▪□□□□▪□▪▪▪▪▪□▪▪▪	777777777413731	1 (0.45)	0.22	EAI1-SOM	Unique
50	▪▪▪▪▪▪▪▪▪▪▪▪▪▪▪▪▪▪▪▪▪▪▪▪▪▪▪▪▪▪□▪□□□□▪▪▪▪▪▪▪	777777777720771	3 (1.36)	0.08	H3	Clustered
51	▪▪▪▪▪▪▪▪▪▪▪▪▪▪▪▪▪▪▪▪▪▪▪▪▪▪▪▪▪▪▪▪□□□□▪▪▪□□□□	777777777760700	2 (0.91)	0.64	T1	Clustered
53	▪▪▪▪▪▪▪▪▪▪▪▪▪▪▪▪▪▪▪▪▪▪▪▪▪▪▪▪▪▪▪▪□□□□▪▪▪▪▪▪▪	777777777760771	1 (0.45)	0.02	T1	Unique
54	▪▪▪▪▪▪▪▪▪▪▪▪▪▪▪▪▪▪▪▪▪▪▪▪▪▪▪▪▪▪▪▪□□▪▪▪▪▪▪▪▪▪	777777777763771	1 (0.45)	0.39	Manu2	Unique
89	▪▪□▪▪▪▪□□□□□□□□□□□□□□□□□□▪▪▪□□□□▪□▪▪▪▪▪▪▪▪▪	674000003413771	5 (2.27)	4.42	EAI2-nonthaburi	Clustered
126	▪□□▪▪▪▪▪▪▪▪▪▪▪▪▪▪▪▪▪▪▪▪▪▪▪▪▪□□□□▪□▪▪▪▪▪▪▪▪▪	477777777413771	1 (0.45)	0.81	EAI5	Unique
236	▪▪▪▪▪▪▪▪▪▪▪▪▪▪▪▪▪▪▪▪▪▪▪▪▪▪▪▪□□□□▪□▪▪▪▪▪▪▪▪▪	777777777413771	10 (4.55)	5.65	EAI5	Clustered
250	□□□□□□□□□□□□□□□□□□□□□□□□□□□□□□□□□□□□□▪▪▪▪▪▪	000000000000371	2 (0.91)	5.26	Beijing	Clustered
256	▪▪▪▪▪▪▪▪▪▪▪▪▪▪▪▪▪▪▪▪▪▪▪▪▪▪▪▪□□□□▪□▪▪▪▪□▪▪▪▪	777777777413671	12 (5.45)	18.18	EAI5	Clustered
260	□□□□□□□□□□□□□□□□□□□□□□□□□□□□□□□□□□▪▪□□▪▪▪▪▪	000000000003171	1 (0.45)	10	Beijing	Unique
298	▪□□▪▪▪▪▪▪▪▪▪▪▪□▪▪▪▪▪▪▪▪▪▪▪▪▪□□□□▪□▪▪□□□▪▪▪▪	477767777413071	1 (0.45)	2.33	EAI3-IND	Unique
340	▪□□▪▪▪▪□□□▪▪▪▪▪▪▪▪▪▪▪▪▪▪▪▪▪▪□□□□▪□▪▪▪▪▪▪▪▪▪	474377777413771	1 (0.45)	2.13	EAI5	Unique
349	▪▪▪▪▪▪▪▪▪▪▪▪□▪▪▪▪▪▪▪▪▪▪▪▪▪▪▪□□□□▪□▪▪▪▪▪□▪▪▪	777737777413731	1 (0.45)	3.45	EAI1-SOM	Unique
374	▪▪▪▪▪▪▪▪▪▪▪▪▪▪▪▪▪▪▪▪▪▪▪▪▪▪□□□□□□□□□□▪▪▪▪▪▪▪	777777776000771	1 (0.45)	11.11	Unknown	Unique
429	▪▪▪□□□□▪▪▪▪▪▪▪▪▪▪▪▪▪▪▪□□□□□□□□□□□□▪▪▪▪▪□▪▪▪	703777740003731	1 (0.45)	3.33	CAS1-Delhi	Unique
483	▪▪□▪▪▪▪▪▪▪▪▪▪▪▪▪▪▪▪□□▪▪▪▪▪▪▪□□□□▪□▪▪▪▪▪□□□▪	677777477413701	1 (0.45)	5	EAI2-Manila	Unique
581	▪▪▪▪▪▪▪▪▪▪▪▪▪▪▪▪▪▪▪▪□▪▪▪▪▪▪▪□▪▪▪□□□□▪▪□▪▪▪▪	777777677560671	1 (0.45)	25	T1	Unique
591	▪▪▪▪▪▪▪▪▪▪▪▪▪▪▪▪▪▪▪▪▪▪□▪▪▪▪▪□□□□▪□▪▪▪▪▪▪▪▪▪	777777757413771	13 (5.91)	14.77	EAI6-BGD1	Clustered
623	▪▪▪▪▪▪▪▪▪▪▪▪▪▪▪▪▪▪▪▪▪▪□▪▪▪▪▪▪▪▪▪▪▪▪▪▪▪▪▪▪▪▪	777777757777771	1 (0.45)	7.14	Unknown	Unique
655	▪□□▪▪▪▪▪▪▪▪▪▪▪▪▪▪▪▪▪▪▪▪▪▪▪▪▪▪▪□▪□□□□▪▪▪▪▪▪▪	477777777720771	2 (0.91)	4.17	H3	Clustered
733	▪▪▪▪▪▪▪▪▪▪▪▪▪▪▪▪□▪▪▪▪▪□□▪▪▪▪□□□□▪□▪▪▪▪▪▪▪▪▪	777775747413771	1 (0.45)	12.5	EAI6-BGD1	Unique
745	▪▪▪▪▪▪▪▪▪▪▪▪▪▪▪▪▪▪▪▪▪▪▪▪▪▪▪▪□□□□▪□▪▪□□▪□▪▪▪	777777777413131	33 (15)	54.1	EAI1-SOM	Clustered
939	▪▪▪▪▪▪▪□▪▪▪▪▪▪▪▪▪▪▪▪▪▪▪▪▪▪▪▪□□□□▪□▪▪▪▪▪▪▪▪▪	775777777413771	1 (0.45)	20	EAI5	Unique
944	▪▪▪▪▪▪▪▪▪▪▪▪▪▪▪▪▪▪▪▪▪▪▪▪▪□□□□□□□□□□□□□□▪▪▪▪	777777774000071	1 (0.45)	20	Unknown	Unique
946	▪▪▪▪▪▪▪▪▪▪▪▪▪▪▪▪▪▪▪▪▪▪□□□□□□□□□▪□□□□▪▪▪▪▪▪▪	777777740020771	1 (0.45)	4.76	H	Unique
951	▪▪▪▪▪▪▪□□□□▪▪▪▪▪▪▪▪▪▪▪▪▪▪▪▪▪□□□□▪□▪▪▪▪▪▪▪▪▪	774177777413771	1 (0.45)	4	EAI5	Unique
962	▪▪▪▪▪▪▪▪▪▪▪▪▪▪▪▪▪▪▪▪▪▪▪▪▪▪▪▪□□□□▪□▪▪□□□□▪▪▪	777777777413031	1 (0.45)	11.11	EAI5	Unique
1183	▪▪▪▪▪▪▪▪▪▪▪▪▪▪▪▪▪▪▪▪▪▪▪▪▪▪▪▪□□□□▪□▪▪□▪▪□▪▪▪	777777777413331	1 (0.45)	25	EAI1-SOM	Unique
1184	□□□□□□□□□□□□□□□□▪□□□□□□□□□□□□□□□□□▪▪▪▪▪▪▪▪▪	000002000003771	1 (0.45)	11.11	Unknown	Unique
1243	▪▪▪▪▪▪▪▪▪□▪▪▪▪▪▪▪▪▪▪▪▪▪▪▪▪▪▪▪▪□▪□□□□▪▪▪▪▪▪▪	777377777720771	1 (0.45)	5.88	H3	Unique
1268	▪▪▪▪▪▪▪▪▪▪▪▪▪▪□□□□□□□▪□▪▪▪▪▪▪▪▪▪□□□□▪▪▪▪▪▪▪	777760057760771	1 (0.45)	11.11	T5	Unique
1372	▪▪▪▪▪▪▪▪▪▪□▪▪▪▪▪▪▪▪▪▪▪▪▪▪▪▪▪□□□□▪□▪▪▪▪▪▪▪▪▪	777577777413771	1 (0.45)	20	EAI5	Unique
1395	▪□□▪▪▪▪□□□▪▪▪▪▪▪▪▪▪▪▪▪▪□▪▪▪▪□□□□▪□▪▪▪▪▪▪▪▪▪	474377767413771	1 (0.45)	16.67	EAI5	Unique
1490	▪▪□▪▪▪▪▪▪▪▪▪▪▪□▪▪▪▪□□▪▪▪▪▪▪▪□□□□▪□▪▪▪▪▪▪▪▪▪	677767477413771	1 (0.45)	16.67	EAI2-Manila	Unique
1494	▪▪▪▪▪▪▪▪▪▪▪▪▪▪▪▪▪▪▪▪□▪▪▪▪▪□▪▪▪▪▪□□□□▪▪▪▪▪▪▪	777777676760771	1 (0.45)	16.67	T1	Unique
1512	▪▪□▪□▪▪▪▪▪▪▪▪▪▪▪▪▪▪□□▪▪▪▪▪▪▪□□□□▪□▪▪▪▪▪□□□▪	657777477413701	1 (0.45)	16.67	EAI2-Manila	Unique
1651	□□□□□□□□□□□□□□□□□□□□□□□□□□□□□□□□□□▪▪▪▪□▪▪□▪	000000000003661	2 (0.91)	12.5	Beijing	Clustered
2383	▪▪▪▪□▪▪▪▪▪▪▪□▪▪▪▪▪▪▪□□□□▪▪▪▪▪▪▪▪□□□□▪▪▪▪▪▪▪	757737607760771	1 (0.45)	16.67	LAM5	Unique
2428	▪▪□▪▪▪▪□□□□□□□▪▪▪▪▪▪□□□□▪▪▪▪▪▪▪▪□□□□▪▪▪▪▪▪▪	674017607760771	1 (0.45)	16.67	LAM3	Unique
2610	□□□□□□□□□□□□□□□□□□□□□□□□□□□□□□□□□□▪▪▪▪▪▪▪▪□	000000000003770	2 (0.91)	28.57	Beijing	Clustered
3143	▪▪▪▪▪▪▪▪▪▪▪▪▪▪□▪▪▪▪▪▪▪▪▪▪▪▪▪▪▪▪▪□□□□▪▪▪□□□□	777767777760700	1 (0.45)	33.33	T1	Unique
3484	▪▪▪□▪▪▪▪▪▪▪▪▪▪▪▪▪▪▪▪▪▪□▪▪▪▪▪□□□□▪□▪▪▪▪▪▪▪▪▪	737777757413771	1 (0.45)	33.33	EAI6-BGD1	Unique
3949	▪□□▪▪▪▪▪▪▪▪▪▪▪▪▪▪▪▪▪▪▪▪▪▪▪▪▪□□□□▪□▪▪□□□□□□▪	477777777413001	1 (0.45)	33.33	EAI5	Unique
3993*	▪▪▪▪▪▪▪▪▪▪▪▪▪▪▪▪▪▪▪▪▪▪▪▪▪▪▪▪□□□□▪□□□□□□□▪▪▪	777777777410031	1 (0.45)	50	EAI5	Unique
3994*	▪▪▪▪▪▪▪▪▪▪▪▪□▪▪▪▪▪▪▪▪▪▪▪▪□▪▪□□□□▪□▪▪▪▪▪□▪▪▪	777737775413731	2 (0.91)	66.67	EAI1-SOM	Clustered
3995*	▪▪▪▪▪▪▪▪□▪▪▪▪▪▪▪▪▪▪▪▪▪▪▪▪▪▪▪□□□□▪□▪▪▪▪▪▪▪▪▪	776777777413771	2 (0.91)	100	EAI5	Clustered
3996*	▪▪▪▪▪▪▪▪▪▪▪▪▪▪▪▪▪▪▪▪□□▪▪▪□□□□□□□□□□□□□□▪▪▪▪	777777634000071	2 (0.91)	100	Unknown	Clustered
3997*	▪▪▪▪▪▪▪▪▪▪▪▪▪▪▪▪▪▪▪▪▪▪▪▪▪▪▪▪□□□□▪□▪▪□□▪□□□▪	777777777413101	2 (0.91)	100	EAI5	Clustered
3998*	▪▪▪▪□□□□□□□□▪▪▪▪▪▪▪▪▪▪□▪▪▪▪▪□□□□▪□▪▪▪▪▪▪▪▪▪	740077757413771	2 (0.91)	100	EAI6-BGD1	Clustered

a Description of 55 shared-types (SITs; n = 198 isolates): 49/55 SITs containing 187 isolates matched a preexisting shared-type in the database, whereas 6/55 SITs (n = 11 isolates) were newly created. A total of 18/55 SITs containing 161 isolates were clustered within this study (2 to 56 isolates per cluster) while 37/55 SITs containing 37 strains were unique (for total unique strains, one should add to this number the 22 orphan strains, which brings the number of unclustered isolates in this study to 59/220 or 26.82%, and clustered isolates to 161/220 or 73.18%). Note that SITs followed by an asterisk indicates "newly created” SITs due to 2 or more strains belonging to an identical new pattern within this study or after a match with an orphan in the database; SIT designations followed by number of strains: 3993* this study n = 1, MYS n = 1; 3994* this study n = 2, MYS n = 1; 3994* this study n = 2; 3995* this study n = 2; 3996* this study n = 2; 3997* this study n = 2; 3998* this study n = 2.

b Lineage designations according to SITVITWEB rules; “Unknown” designates patterns with signatures that do not belong to any of the major lineages described in the database.

c Clustered strains correspond to a similar spoligotype pattern shared by 2 or more strains “within this study”; as opposed to unique strains harboring a spoligotype pattern that does not match with another strain from this study. Unique strains matching a preexisting pattern in the SITVIT2 database are classified as SITs, whereas in case of no match, they are designated as “orphan” (see Table 1).

The eight most common SITs grouped 141 isolates (**Table 2**; 3 to 56 strains per SIT): SIT1/Beijing, n = 56, 25.5%; SIT745/EAI1-SOM, n = 33, 15.0%; SIT591/EAI6-BGD1, n = 13, 5.9%; SIT256/EAI5, n = 12, 5.5%; SIT236/EAI5, n = 10, 4.6%; SIT19/EAI2-Manila, n = 9, 4.1%; SIT89/EAI2-Nonthaburi, n = 5, 2.3%; and SIT50/H3, n = 3, 1.4%. A careful inspection of their worldwide distribution in countries and regions with ≥3% of a given SIT (**Table 2**) clearly highlighted the fact that all of them, with the exception of SIT50/H3, showed a remarkable phylogeographical predominance in South East-Asia, followed by in South or East-Asia.

**Table 2:** Description of clusters containing >1% (n = 3 or more) isolates in this study and their worldwide distribution in the SITVIT2 database.

SIT (Lineage) Octal Number Spoligotype Description	Nb (%) in study	% in study vs. database	Distribution in Regions with ≥3% of a given SIT a	Distribution in countries with ≥3% of a given SIT b
1 (Beijing) 00000000000377 □□□□□□□□□□□□□□□□□□□□□□□□□□□□□□□□□□▪▪▪▪▪▪▪▪▪	56 (25.45)	0.54	ASIA-E 31.74, AMER-N 19.4, ASIA-SE 10.31, AFRI-S 8.01, ASIA-N 6.72, ASIA-S 5.16, EURO-N 3.53, AMER-S 3.47	USA 19.16, CHN 18.34, JPN 11.13, ZAF 8.01, RUS 6.72, VNM 3.77, PER 3.07
745 (EAI1-SOM) 777777777413131 ▪▪▪▪▪▪▪▪▪▪▪▪▪▪▪▪▪▪▪▪▪▪▪▪▪▪▪▪□□□□▪□▪▪□□▪□▪▪▪	33 (15)	54.1	ASIA-SE 96.72, ASIA-S 3.28	MYS 96.72, IND 3.28
591 (EAI6-BGD1) 777777757413771 ▪▪▪▪▪▪▪▪▪▪▪▪▪▪▪▪▪▪▪▪▪▪□▪▪▪▪▪□□□□▪□▪▪▪▪▪▪▪▪▪	13 (5.91)	14.77	ASIA-SE 35.23, ASIA-S 22.73, EURO-N 21.59, EURO-W 9.09, ASIA-W 6.82	MYS 26.14, SWE 17.05, IND 11.36, BGD 11.36, MMR 5.68, OMN 4.55, NLD 4.55
256 (EAI5) 777777777413671 ▪▪▪▪▪▪▪▪▪▪▪▪▪▪▪▪▪▪▪▪▪▪▪▪▪▪▪▪□□□□▪□▪▪▪▪□▪▪▪▪	12 (5.45)	18.18	ASIA-SE 68.18, AMER-N 18.18, ASIA-W 4.55, ASIA-S 3.03	MYS 45.46, USA 18.18, THA 15.15, SAU 4.55, MMR 3.03, IND 3.03, IDN 3.03
236 (EAI5) 777777777413771 ▪▪▪▪▪▪▪▪▪▪▪▪▪▪▪▪▪▪▪▪▪▪▪▪▪▪▪▪□□□□▪□▪▪▪▪▪▪▪▪▪	10 (4.55)	5.65	AMER-N 25.99, ASIA-SE 23.16, ASIA-S 22.03, ASIA-W 8.48, EURO-N 6.22, EURO-W 5.65, AUST 3.96	USA 25.99, IND 12.99, MYS 9.61, BGD 8.48, THA 6.78, SAU 5.09, MMR 3.39, FXX 3.39
19 (EAI2-Manila) 677777477413771 ▪▪□▪▪▪▪▪▪▪▪▪▪▪▪▪▪▪▪□□▪▪▪▪▪▪▪□□□□▪□▪▪▪▪▪▪▪▪▪	9 (4.09)	0.99	AMER-N 45.66, ASIA-SE 20.9, AMER-C 9.13, ASIA-E 6.71, EURO-S 4.29, ASIA-W 4.29, EURO-W 3.08	USA 39.38, PHL 12.1, MEX 8.58, MYS 5.72, ITA 4.29, TWN 3.96, SAU 3.19
89 (EAI2-Nonthaburi) 674000003413771 ▪▪□▪▪▪▪□□□□□□□□□□□□□□□□□□▪▪▪□□□□▪□▪▪▪▪▪▪▪▪▪	5 (2.27)	4.42	ASIA-SE 53.1, AMER-N 18.58, EURO-N 7.97, EURO-W 6.2, ASIA-W 4.43, AMER-S 3.54	THA 31.86, USA 18.58, MMR 8.85, MYS 7.97, SWE 4.43, NLD 4.43, SAU 3.54, FIN 3.54
50 (H3) 777777777720771 ▪▪▪▪▪▪▪▪▪▪▪▪▪▪▪▪▪▪▪▪▪▪▪▪▪▪▪▪▪▪□▪□□□□▪▪▪▪▪▪▪	3 (1.36)	0.08	AMER-S 25.37, EURO-W 15.13, AMER-N 15.13, EURO-S 9.94, CARI 5.01, EURO-E 4.75, EURO-N 4.7, AFRI-N 3.66, AFRI-S 3.48, AFRI-M 3.25	USA 15.1, PER 13.78, BRA 6.48, FXX 5.92, AUT 5.24, ITA 4.68, ESP 4.68, ZAF 3.48, CMR 3.2, CZE 3.15

a Worldwide distribution is reported for regions with more than 3% of a given SITs as compared to their total number in the SITVIT2 database. The definition of macro-geographical regions and sub-regions (http://unstats.un.org/unsd/methods/m49/m49regin.htm) is according to the United Nations; Regions: AFRI (Africa), AMER (Americas), ASIA (Asia), EURO (Europe), and OCE (Oceania), subdivided in: E (Eastern), M (Middle), C (Central), N (Northern), S (Southern), SE (South-Eastern), and W (Western). Furthermore, CARIB (Caribbean) belongs to Americas, while Oceania is subdivided in 4 sub-regions, AUST (Australasia), MEL (Melanesia), MIC (Micronesia), and POLY (Polynesia). Note that in our classification scheme, Russia has been attributed a new sub-region by itself (Northern Asia) instead of including it among rest of the Eastern Europe. It reflects its geographical localization as well as due to the similarity of specific TB genotypes circulating in Russia (a majority of Beijing genotypes) with those prevalent in Central, Eastern and South-Eastern Asia.

b The 3 letter country codes are according to http://en.wikipedia.org/wiki/ISO_3166-1_alpha-3; countrywide distribution is only shown for SITs with ≥3% of a given SITs as compared to their total number in the SITVIT2 database.

Regarding clustering, a total of 18/55 SITs containing 161 isolates were clustered within this study (2 to 56 isolates per cluster) while 37/55 SITs containing 37 strains were unique (for total unique strains, one should add to this number the 22 orphan strains, which brings the number of unclustered isolates in this study to 59/220 or 26.82%, and clustered isolates to 161/220 or 73.18%).

If the study sample was classified by clades (and not SITs), the predominant *M. tuberculosis* clades belonged to the ancestral EAI clade (56.4%) followed by Beijing (28.6%), Haarlem (4.0%) and T (4.0%). The single *M. bovis* clade identified in this study was isolated from a 14 year old Malay boy with pulmonary TB; unfortunately, DST patterns were not available for this strain. **Table 3** summarizes available demographic, epidemiologic and DST results in function of clades, and one may notice that the proportion of drug resistant strains was higher among isolates belonging to the Beijing lineage (8 drug resistant isolates vs. 29 pansusceptible isolates) as compared to the isolates belonging to EAI lineage (9 drug resistant isolates vs. 83 pansusceptible isolates). Apart from that, almost all clades were reported higher in male group than female group except for H clade. Nevertheless, statistical analyses of *M. tuberculosis* clades by patient demographic and drug resistance criteria showed that neither differences observed between gender and clades nor those between drug resistance and clades were statistically significant (p>0.5). On the other hand, the association between city of isolation and clades was statistically significant; the proportion of Haarlem and T lineages being higher in Kuala Lumpur than Kelantan (p<0.01).

**Table 3:** *M. tuberculosis* clade by patient demographic and drug resistance criteria.

Clade	Female	Male	M/F Sex ratio	Mean age	Kelantan	Kuala Lumpur	Unknown Drug R	Pan-susceptible	Drug resistant	Total (%)
**BOV**	0	1	-	14	1	0	1	0	0	1 (0.5)
**Beijing**	24	39	1.63	47.67	47	16	26	29	8	63 (28.6)
**CAS**	1	1	1.00	40	0	2	2	0	0	2 (0.9)
**EAI**	46	78	1.70	51.45	116	8	32	83	9	124 (56.4)
**H**	5	4	0.80	46.33	5	4	7	2	0	9 (4.1)
**LAM**	1	1	1.00	31	2	0	0	1	1	2 (0.9)
**Manu**	0	1	-	46	1	0	1	0	0	1 (0.5)
**S**	0	1	-	63	1	0	0	1	0	1 (0.5)
**T**	3	6	2.00	45.11	4	5	7	2	0	9 (4.1)
**Unknown**	2	6	3.00	44.5	7	1	1	7	0	8 (3.6)
**Total**	**82**	**138**	**1.68**	**49.21**	**184**	**36**	**77**	**125**	**18**	**220 (100)**

**Figure 1:**
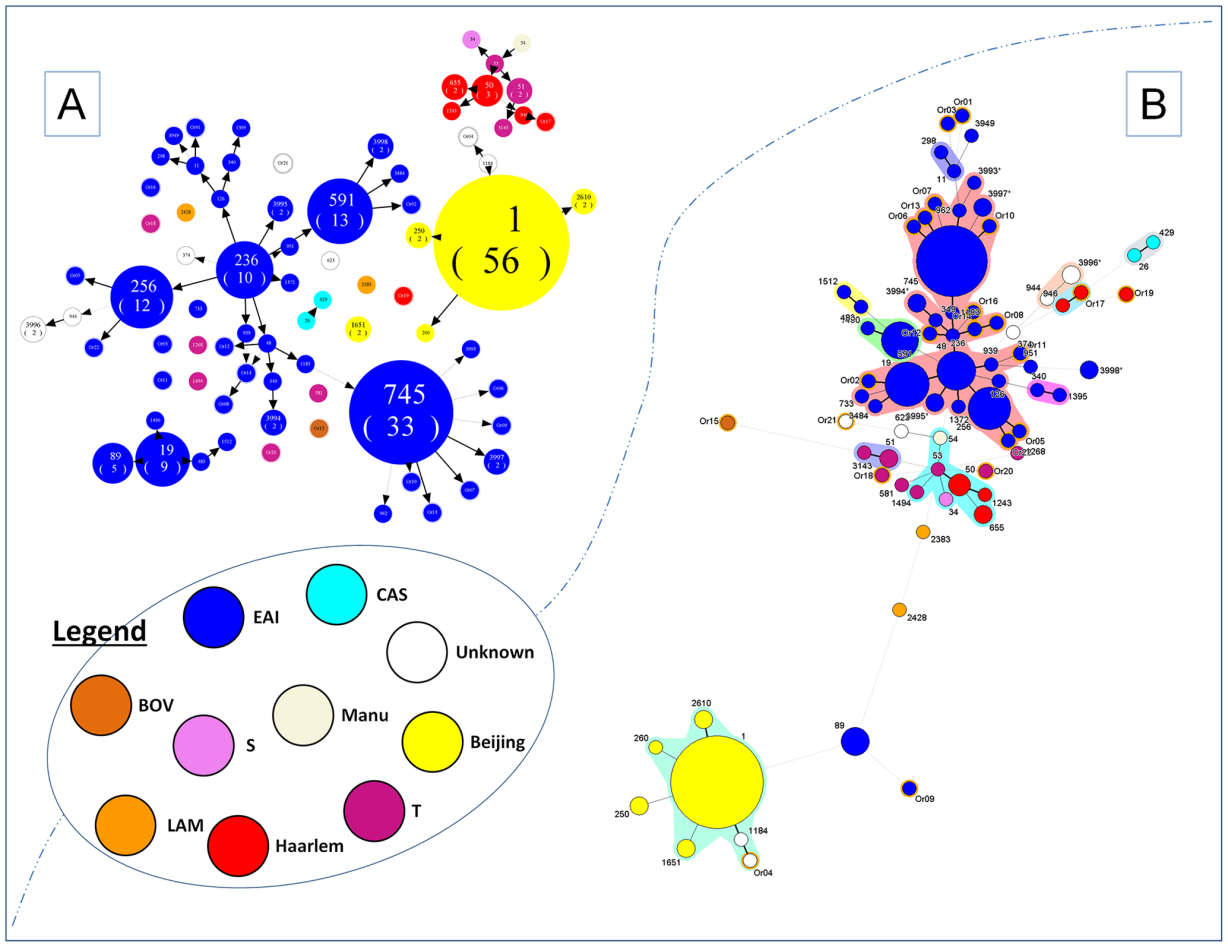
Phylogenetical trees illustrating evolutionary relationships between *M. tuberculosis* spoligotypes in Malaysia. (A) Spoligoforest tree drawn using the SpolTools software (available through http://www.emi.unsw.edu.au/spolTools [14], [15]), and shown as Fruchterman Reingold tree. The Figure was drawn on all patterns including orphan patterns (n = 220). In both cases, each spoligotype pattern from the study is represented by a node with area size being proportional to the total number of isolates with that specific pattern. Changes (loss of spacers) are represented by directed edges between nodes, with the arrowheads pointing to descendant spoligotypes. In this representation, the heuristic used selects a single inbound edge with a maximum weight using a Zipf model. Solid black lines link patterns that are very similar, i.e., loss of one spacer only (maximum weigh being 1.0), while dashed lines represent links of weight comprised between 0.5 and 1, and dotted lines a weight less than 0.5. One may notice the predominance of SIT1/Beijing (n = 56), SIT745/EAI1-SOM (n = 33), SIT591/EAI6-BGD1 (n = 13), SIT256/EAI5 (n = 12), and SIT236/EAI5 (n = 10). Furthermore, note the phylogeographical specificity of SIT745 for Malaysia, and the fact that it seems to have not largely spread in other countries/regions (see Table 3). (B) A minimum spanning tree (MST) illustrating evolutionary relationships between M. tuberculosis spoligotypes. MST constructed on all isolates including the orphan patterns (n = 220). The phylogenetic tree connects each genotype based on degree of changes required to go from one allele to another. The structure of the tree is represented by branches (continuous vs. dashed and dotted lines) and circles representing each individual pattern. Note that the length of the branches represents the distance between patterns while the complexity of the lines (continuous, gray dashed and gray dotted) denotes the number of allele/spacer changes between two patterns: solid lines, 1 or 2 or 3 changes (thicker ones indicate a single change, while the thinner ones indicate 2 or 3 changes); gray dashed lines represent 4 changes; and gray dotted lines represent 5 or more changes. The size of the circle is proportional to the total number of isolates in our study, illustrating unique isolates (smaller nodes) versus clustered isolates (bigger nodes). The color of the circles indicates the phylogenetic lineage to which the specific pattern belongs. Note that orphan patterns are circled in orange (22 strains belonged to orphans in our study). Note that the colors used are the same in Spoligoforest tree and MST.

Investigation of phylogenetic relationships between MTBC strains by Spoligoforest and minimum spanning tree (MST) is shown in **Figs. 1A and 1B**, respectively. The arrowheads in the Spoligoforest point to descendant spoligotypes and the tree provides an overview of the parental links that probably exist between strains belonging to different sublineages as a “directed graph”, while the “undirected network” in MST shows isolates linked together with the fewest possible linkages between nearest neighbors. In our study, both trees highlight that SIT1 (n = 56) constituted the largest node, followed by SIT745 (n = 33), SIT591 (n = 13), SIT256 (n = 12), SIT236 (n = 10), SIT19 (n = 9), SIT89 (n = 5), SIT50 (n = 3), and many smaller nodes containing 2 strains or less. The majority of ancestral EAI strains (shown in deep blue) occupied central positions on the trees, and were linked within a parental network with evidence of ongoing local evolution (in particular for SIT745 at the bottom right position). On the contrary, the rare LAM, T and CAS strains were isolated, suggesting that these strains probably arrived not very long ago. However, a small though closely connected network was visible in case for Haarlem strains (shown in red), suggesting ongoing evolution and local adaptation of these strains, an observation that calls for vigilance.

Another interesting observation was the fact that Beijing family group (shown in yellow) was independent from the central network of EAI lineage strains in Spoligoforest (**Fig. 1A**), and at the very far bottom left of the MST (**Fig. 1B**). One may note the presence of the only two strains belonging to EAI lineage in its vicinity (SIT89/EAI2-Nonthaburi and an orphan strain: Or09/EAI1-SOM). The fact that Beijing strains were isolated without interconnections with other MTBC strains simply reflects the fact that the Beijing lineage has only rare or no recent evolutionary connections with ancestral EAI strains that predominate in South-East Asia. Furthermore, SIT1/Beijing was the most predominant single SIT involved in ongoing TB epidemic in Malaysia, but with very few locally evolved Beijing variants.

**Figure 2:**
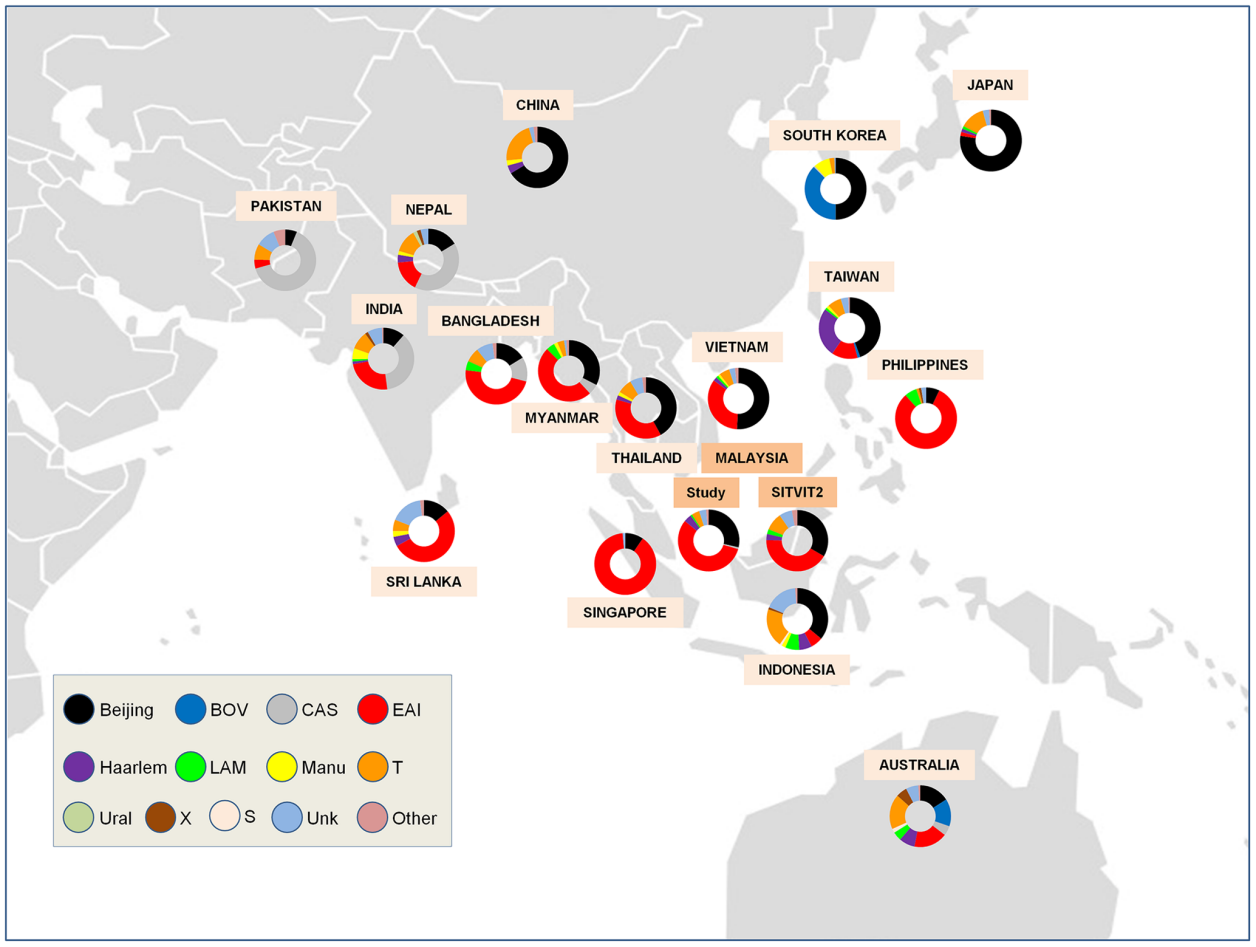
Customized map of phylogeographical distribution of major *M. tuberculosis* lineages in the neighboring countries of Malaysia (data extracted from the updated version of the SITVIT2 database). One may notice the similarity in the lineages distribution of this study as compared to Malaysian strains previously entered in SITVIT2. Note that the map file was downloaded under Creative Commons License using the link: http://upload.wikimedia.org/wikipedia/commons/c/c2/Blank Map Pacific World.svg and was manually modified for representative purposes only.

**Figure 3:**
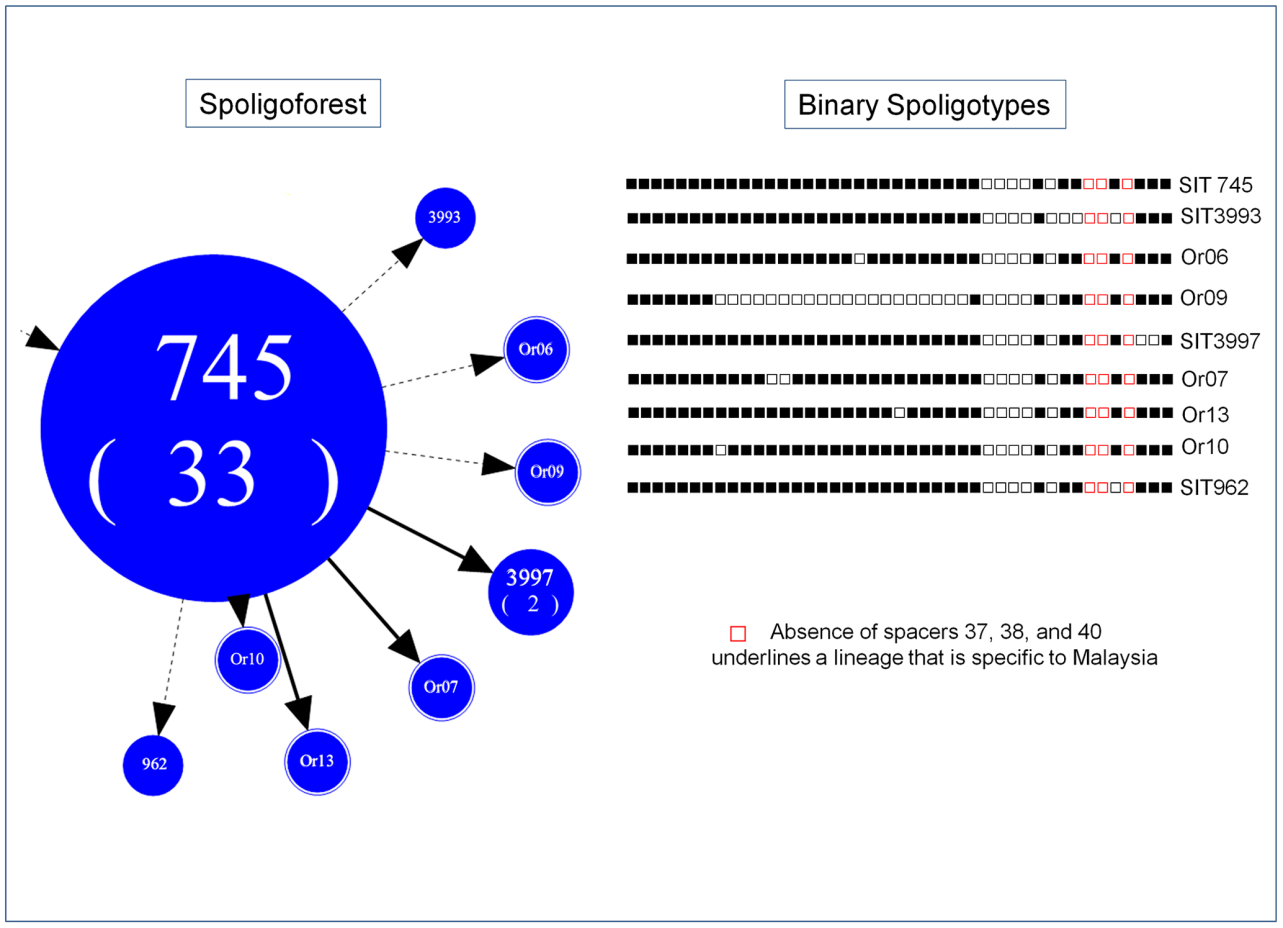
Zoomed part of Spoligoforest tree showing SIT745 tentatively relabeled "EAI1-MYS" and its spoligotype descendants, along with their binary spoligotyping descriptions highlighting the specific absence of spacers 37, 38 and 40.

The map of phylogeographical distribution of major *M. tuberculosis* lineages in Malaysia and its neighboring countries (**Fig. 2**) highlighted that EAI lineage is the most prevalent lineage in Malaysia, Philippines, Singapore, Myanmar, Thailand, Bangladesh and Sri Lanka, while the Beijing lineage is the most prevalent in China, Japan, South Korea, Taiwan, Vietnam and Thailand. Interestingly, the MTBC population structure observed for Malaysia in the present study is almost similar to the one recorded for a total of 749 MTBC strains recorded in the SITVIT2 database. Last but not least, particularly noticeable was the ongoing evolution of the SIT745 in Malaysia which was closely connected to a network of 8 other derived spoligotype patterns (**Fig. 3**), all showing a remarkable phylogeographical specificity for Malaysia. The parental SIT745 pattern and the locally evolved descendant strains shared a specific signature characterized by absence of spacers 37, 38, and 40. Hence pending complementary genotyping confirmation by additional genotypic markers, we propose that SIT745/EAI-SOM is tentatively reclassified as SIT745/EAI-MYS.

## Discussion

This study described the spoligotypes of 220 *M. tuberculosis* isolates from two different geographical areas in Malaysia during a five year period. The SIT1/Beijing spoligotype was recorded as the most predominant strain with 25.5% of isolates belonging to this specific genotype. This finding is consistent with a previous IS*6110*-RFLP study from Malaysia in 1999 [3]. However, the proportion of Beijing genotype is higher in our study as compared to 19.2% reported earlier. It is postulated that, the geographical location of Kelantan beside Thailand, with high proportion of Beijing genotype [17] may have contributed to this finding.

First described in 1995, Beijing genotype shows high prevalence in East-Asian countries [18], representing more than 50% of the isolated strains in certain areas and/or subpopulations; e.g., it accounted for 70% of TB cases in Hong Kong [19], 80.4% in Jiangsu province, China [20], and 77.7% among homeless TB patients in Osaka City, Japan [21]. A similar distribution pattern was also confirmed for China, Japan and Vietnam in the SITVIT2 database (**Fig. 2**). In contrast, the prevalence of the Beijing genotype is low in Latin America as compared to Asian and East-European countries [22]–[27]. Although the Beijing genotype strains are strongly associated with drug resistance and specifically multiple drug resistant (MDR) and extensively drug resistant (XDR) TB in various geographical settings [20], [28]–[30], this association holds true particularly in areas where the proportion of the Beijing genotype strain is actively increasing (reviewed in [31] and references therein).

The single reported isolate of MDR-TB in our study was isolated from a 61 year old woman, and classified as EAI6-BGD1 sublineage. This finding does not waive the probable association of Beijing genotypes with MDR-TB observed elsewhere, since MDR-TB is not yet a serious problem in Malaysia (prevalence of about 0.1%; see reference [32]). In our study, we did not observe a significant association between drug resistance and various clades of *M. tuberculosis*, most probably owing to the moderate drug-resistance observed (**Table 3**). On the other hand, a significant association between the city of isolation and clades was found for Haarlem and T lineages whose proportion was higher in Kuala Lumpur than Kelantan (p<0.01). In addition to the fact that Haarlem and T family isolates are intermittently seen in patients born in Southeast Asia [30], this is most probably linked to a more cosmopolitan nature of Kuala Lumpur with a higher population of travelers and migrants. Lastly, although not statistically significant, the CAS strains were exclusively found in Kuala Lumpur, and are probably linked to migrants from the Indian subcontinent (India, Pakistan, Bangladesh, Nepal) which form an important minority in the capital city. It was previously reported that foreign-born TB patients were more often associated with poor compliance to anti-tuberculosis therapy in Malaysia, and presence of highly mobile illegal migrants, both within the country as well as across borders, further complicated the national tuberculosis program [33].

The spoligotype SIT745 (assigned EAI1-SOM sublineage in SITVIT2, but tentatively relabeled EAI1-MYS in this paper) was the second most predominant pattern in our study. This spoligotype with its descendants (**Fig. 3**) seems to be specific for Malaysia since it is not widely spread in other countries, and may represent a new EAI sublineage with high phylogeographical specificity for Malaysia. Interestingly, SIT745/EAI1-MYS sublineage is tentatively identifiable by the absence of spacers 37 and 38 (a signature common to EAI3-IND sublineage) in conjunction with the absence of spacer 40 (a signature common to EAI1-SOM sublineage). In SITVIT2 database, such strains have only been reported from Malaysia and India, although majorities (>95%) are reported in Malaysia (**Table 2**). Now such strains should be ideally reconfirmed using supplementary genotyping markers and compared to the prototypes of EAI1-SOM and EAI3-IND sublineages to corroborate our findings. This should not come as a surprise since the endemic nature of EAI lineage in Southeast Asia has been well proven with the existence of specific sublineages, e.g. EAI2-Manila with phylogeographical specificity to the Philippines and EAI4-VNM to Vietnam [10]. If proven in future investigations, our study would have the merit to underline the predominance of SIT745/EAI1-MYS sublineage as being specific to Malaysia. It is suggested that there are some specific factors contributing to the current finding and further study is needed to explore it.

Interestingly, the phylogenetical trees which provide a good indication on the parental links that exist between strains belonging to different lineages [24], showed in our case that Beijing, CAS and Haarlem strains were not located at the central positions on the Spoligoforest and MST illustrations (**Fig. 1**). On the other hand, a majority of EAI strains occupied central positions on these trees, and were linked within a parental network with evidence of ongoing local evolution, in particular for SIT745 at the bottom right position in **Fig. 1A**). EAI is the predominant lineage in Malaysia, identified in more than half of overall strains followed by Beijing lineage (**Table 3**). This finding is in line with the previous data from Malaysia reported in the SITVIT2 database (**Fig. 2**). It shows that EAI lineage has high transmission of TB in our study population. One may notice the majority or orphan strains in this study were emerging from this lineage (**Fig. 1**). Even though EAI lineage was the most predominant strain in this study, there was no significant association with gender and drug susceptibility pattern. However, a study in Montreal reported that EAI lineage was associated with lower rates of TB transmission and less likely to cause severe forms of TB, though it was associated with a higher proportion of TB lymphadenitis and extra-pulmonary TB [5]. Apart from that, a study had reported a strong association between patient's country of origin and lineages, where Beijing and EAI families dominated in patients born in Southeast Asia [30].

In conclusion, this preliminary study showed the usefulness and suitability of spoligotyping as a first-line genotyping tool for studying *M. tuberculosis* genotypic diversity in Malaysia. It should now be ideally completed by future prospective studies with inclusion of detailed clinical, demographic, epidemiologic, and drug-susceptibility data in a systematic manner. Such studies would obviously benefit from MIRU-VNTRs analysis for a better discrimination of the MTBC strains, and to identify probable new sublineage(s). Furthermore, these findings could help for diagnostic test development towards targeting critical virulence factors and to identify person at risk for developing active disease thereby limiting disease transmission and the spread of the TB epidemic.

## Supporting Information

Table S1 **Detailed results obtained including demographic, epidemiologic, drug-resistance, and genotyping information on a total of 220** ***M. tuberculosis*** **strains isolated from patients residing in Kelantan (n = 184 strains) and Kuala Lumpur (n = 36), Malaysia.** ^a^ Note that for orphan spoligotype patterns (n = 22 strains marked as Or01-Or22, highlighted in blue), lineages were assigned manually by Expert-based interpretations using SITVITWEB rules. ^b^ Note that SITs followed by an asterisk (n = 6 patterns containing 11 strains, highlighted in yellow) indicate "newly created shared-types" due to 2 or more strains belonging to an identical new pattern within this study or after a match with an orphan in the database; SIT designations followed by number of strains: 3993* this study n = 1, MYS n = 1; 3994* this study n = 2, MYS n = 1; 3995* this study n = 2; 3996* this study n = 2; 3997* this study n = 2; 3998* this study n = 2. ^c^ Drug resistance code; “0” for unknown; “1” for strain susceptible to all first-line drugs; “2” for MDR-TB (combined resistance to INH-RIF); “3” for any other resistances (followed by the name of the drugs: STR, Streptomycin; INH, Isoniazid; RIF, Rifampin; ETB, Ethambutol; PZA, Pyrazinamide).
(PDF)
