# Supplementary material for: Study of Mycobacterium tuberculosis Complex Genotypic Diversity in Malaysia Reveals a Predominance of Ancestral East-African-Indian Lineage with a Malaysia-Specific Signature
Source: PLoS One. 2014 Dec 11;9(12):e114832. doi: 10.1371/journal.pone.0114832 (PMC4263714; doi:10.1371/journal.pone.0114832)
Supplement: Table S1 — Detailed results obtained including demographic, epidemiologic, drug-resistance, and genotyping information on a total of 220 M. tuberculosis strains isolated from patients residing in Kelantan (n = 184 strains) and Kuala Lumpur (n = 36), Malaysia. a Note that for orphan spoligotype patterns (n = 22 strains marked as Or01-Or22, highlighted in blue), lineages were assigned manually by Expert-based interpretations using SITVITWEB rules. b Note that SITs followed by an asterisk (n = 6 patterns containing 11 strains, highlighted in yellow) indicate "newly created shared-types" due to 2 or more strains belonging to an identical new pattern within this study or after a match with an orphan in the database; SIT designations followed by number of strains: 3993* this study n = 1, MYS n = 1; 3994* this study n = 2, MYS n = 1; 3995* this study n = 2; 3996* this study n = 2; 3997* this study n = 2; 3998* this study n = 2. c Drug resistance code; “0” for unknown; “1” for strain susceptible to all first-line drugs; “2” for MDR-TB (combined resistance to INH-RIF); “3” for any other resistances (followed by the name of the drugs: STR, Streptomycin; INH, Isoniazid; RIF, Rifampin; ETB, Ethambutol; PZA, Pyrazinamide). (PDF) [file pone.0114832.s001.pdf]

Supplemental Table S1. Detailed results obtained including demographic, epidemiologic, drug-resistance, and genotyping information on a total of 220 *M. tuberculosis* strains isolated from patients residing in Kelantan (n=184 strains) and Kuala Lumpur (n=36), Malaysia.

Strain ID	Year	Isolation city	Sex/ Age	Spoligotype Description	SIT [a, b]	Lineage	DST code [c] (comments)
00001	2010	Kelantan	M/65	□□□□□□□□□□□□□□□□□□□□□□□□□□□□□□□□□□■■■■■■■■■	1	Beijing	1
00011	2011	Kelantan	M/61	□□□□□□□□□□□□□□□□□□□□□□□□□□□□□□□□□□■■■■■■■■■	1	Beijing	1
00015	2011	Kelantan	M/30	□□□□□□□□□□□□□□□□□□□□□□□□□□□□□□□□□□■■■■■■■■■	1	Beijing	1
00016	2011	Kelantan	F/58	□□□□□□□□□□□□□□□□□□□□□□□□□□□□□□□□□□■■■■■■■■■	1	Beijing	1
00017	2011	Kelantan	F/54	□□□□□□□□□□□□□□□□□□□□□□□□□□□□□□□□□□■■■■■■■■■	1	Beijing	0
00026	2011	Kelantan	M/45	□□□□□□□□□□□□□□□□□□□□□□□□□□□□□□□□□□■■■■■■■■■	1	Beijing	1
00035	2011	Kelantan	M/72	□□□□□□□□□□□□□□□□□□□□□□□□□□□□□□□□□□■■■■■■■■■	1	Beijing	1
00037	2011	Kelantan	M/52	□□□□□□□□□□□□□□□□□□□□□□□□□□□□□□□□□□■■■■■■■■■	1	Beijing	1
00040	2011	Kelantan	F/62	□□□□□□□□□□□□□□□□□□□□□□□□□□□□□□□□□□■■■■■■■■■	1	Beijing	0
00043	2011	Kelantan	M/41	□□□□□□□□□□□□□□□□□□□□□□□□□□□□□□□□□□■■■■■■■■■	1	Beijing	1
00052	0000	Kelantan	M/40	□□□□□□□□□□□□□□□□□□□□□□□□□□□□□□□□□□■■■■■■■■■	1	Beijing	3 (INH-R, STR-R)
00056	2011	Kelantan	F/23	□□□□□□□□□□□□□□□□□□□□□□□□□□□□□□□□□□■■■■■■■■■	1	Beijing	1
00057	2011	Kelantan	M/35	□□□□□□□□□□□□□□□□□□□□□□□□□□□□□□□□□□■■■■■■■■■	1	Beijing	3 (RIF-R)
00061	2012	Kelantan	M/18	□□□□□□□□□□□□□□□□□□□□□□□□□□□□□□□□□□■■■■■■■■■	1	Beijing	1
00063	2010	Kelantan	M/54	□□□□□□□□□□□□□□□□□□□□□□□□□□□□□□□□□□■■■■■■■■■	1	Beijing	3 (STR-R)
00075	2012	Kelantan	F/53	□□□□□□□□□□□□□□□□□□□□□□□□□□□□□□□□□□■■■■■■■■■	1	Beijing	1
00079	2012	Kelantan	M/64	□□□□□□□□□□□□□□□□□□□□□□□□□□□□□□□□□□■■■■■■■■■	1	Beijing	1
00084	2012	Kelantan	M/45	□□□□□□□□□□□□□□□□□□□□□□□□□□□□□□□□□□■■■■■■■■■	1	Beijing	1
00087	2011	Kelantan	M/62	□□□□□□□□□□□□□□□□□□□□□□□□□□□□□□□□□□■■■■■■■■■	1	Beijing	3 (RIF-R)
00089	2012	Kelantan	M/62	□□□□□□□□□□□□□□□□□□□□□□□□□□□□□□□□□□■■■■■■■■■	1	Beijing	3 (RIF-R)
00093	2012	Kelantan	F/16	□□□□□□□□□□□□□□□□□□□□□□□□□□□□□□□□□□■■■■■■■■■	1	Beijing	1
00094	2012	Kelantan	F/55	□□□□□□□□□□□□□□□□□□□□□□□□□□□□□□□□□□■■■■■■■■■	1	Beijing	1
00096	2012	Kelantan	M/74	□□□□□□□□□□□□□□□□□□□□□□□□□□□□□□□□□□■■■■■■■■■	1	Beijing	1
00103	2012	Kelantan	M/67	□□□□□□□□□□□□□□□□□□□□□□□□□□□□□□□□□□■■■■■■■■■	1	Beijing	1
00104	2012	Kelantan	F/24	□□□□□□□□□□□□□□□□□□□□□□□□□□□□□□□□□□■■■■■■■■■	1	Beijing	3 (INH-R)
00105	2012	Kelantan	M/36	□□□□□□□□□□□□□□□□□□□□□□□□□□□□□□□□□□■■■■■■■■■	1	Beijing	1
00106	2012	Kelantan	M/34	□□□□□□□□□□□□□□□□□□□□□□□□□□□□□□□□□□■■■■■■■■■	1	Beijing	3 (STR-R)
00110	2012	Kelantan	M/39	□□□□□□□□□□□□□□□□□□□□□□□□□□□□□□□□□□■■■■■■■■■	1	Beijing	1
00112	2012	Kelantan	M/53	□□□□□□□□□□□□□□□□□□□□□□□□□□□□□□□□□□■■■■■■■■■	1	Beijing	1
00113	2012	Kelantan	F/23	□□□□□□□□□□□□□□□□□□□□□□□□□□□□□□□□□□■■■■■■■■■	1	Beijing	1
00120	2013	Kelantan	F/50	□□□□□□□□□□□□□□□□□□□□□□□□□□□□□□□□□□■■■■■■■■■	1	Beijing	3 (STR-R)
00126	2013	Kelantan	F/19	□□□□□□□□□□□□□□□□□□□□□□□□□□□□□□□□□□■■■■■■■■■	1	Beijing	1
00129	2013	Kelantan	F/42	□□□□□□□□□□□□□□□□□□□□□□□□□□□□□□□□□□■■■■■■■■■	1	Beijing	1
00134	2013	Kelantan	F/19	□□□□□□□□□□□□□□□□□□□□□□□□□□□□□□□□□□■■■■■■■■■	1	Beijing	1
00138	2013	Kelantan	F/60	□□□□□□□□□□□□□□□□□□□□□□□□□□□□□□□□□□■■■■■■■■■	1	Beijing	1
00144	2013	Kelantan	M/40	□□□□□□□□□□□□□□□□□□□□□□□□□□□□□□□□□□■■■■■■■■■	1	Beijing	0
00147	2013	Kelantan	F/43	□□□□□□□□□□□□□□□□□□□□□□□□□□□□□□□□□□■■■■■■■■■	1	Beijing	1
00148	2013	Kelantan	M/74	□□□□□□□□□□□□□□□□□□□□□□□□□□□□□□□□□□■■■■■■■■■	1	Beijing	1
00150	2013	Kelantan	M/52	□□□□□□□□□□□□□□□□□□□□□□□□□□□□□□□□□□■■■■■■■■■	1	Beijing	0
00157	2013	Kelantan	M/70	□□□□□□□□□□□□□□□□□□□□□□□□□□□□□□□□□□■■■■■■■■■	1	Beijing	0
00170	2011	Kelantan	M/56	□□□□□□□□□□□□□□□□□□□□□□□□□□□□□□□□□□■■■■■■■■■	1	Beijing	0
00182	2011	Kelantan	F/66	□□□□□□□□□□□□□□□□□□□□□□□□□□□□□□□□□□■■■■■■■■■	1	Beijing	0
00183	2013	Kelantan	F/49	□□□□□□□□□□□□□□□□□□□□□□□□□□□□□□□□□□■■■■■■■■■	1	Beijing	0
00186	2013	Kuala Lumpur	F/29	□□□□□□□□□□□□□□□□□□□□□□□□□□□□□□□□□□■■■■■■■■■	1	Beijing	0
00187	2013	Kuala Lumpur	M/54	□□□□□□□□□□□□□□□□□□□□□□□□□□□□□□□□□□■■■■■■■■■	1	Beijing	0
00193	2013	Kuala Lumpur	M/61	□□□□□□□□□□□□□□□□□□□□□□□□□□□□□□□□□□■■■■■■■■■	1	Beijing	0
00195	2013	Kuala Lumpur	F/19	□□□□□□□□□□□□□□□□□□□□□□□□□□□□□□□□□□■■■■■■■■■	1	Beijing	0
00197	2013	Kuala Lumpur	M/56	□□□□□□□□□□□□□□□□□□□□□□□□□□□□□□□□□□■■■■■■■■■	1	Beijing	0
00198	2013	Kuala Lumpur	M/42	□□□□□□□□□□□□□□□□□□□□□□□□□□□□□□□□□□■■■■■■■■■	1	Beijing	0
00199	2013	Kuala Lumpur	M/52	□□□□□□□□□□□□□□□□□□□□□□□□□□□□□□□□□□■■■■■■■■■	1	Beijing	0
00200	2013	Kuala Lumpur	M/61	□□□□□□□□□□□□□□□□□□□□□□□□□□□□□□□□□□■■■■■■■■■	1	Beijing	0
00201	2013	Kuala Lumpur	F/26	□□□□□□□□□□□□□□□□□□□□□□□□□□□□□□□□□□■■■■■■■■■	1	Beijing	0
00209	2013	Kuala Lumpur	M/47	□□□□□□□□□□□□□□□□□□□□□□□□□□□□□□□□□□■■■■■■■■■	1	Beijing	0
00210	2013	Kuala Lumpur	M/47	□□□□□□□□□□□□□□□□□□□□□□□□□□□□□□□□□□■■■■■■■■■	1	Beijing	0
00213	2013	Kuala Lumpur	F/19	□□□□□□□□□□□□□□□□□□□□□□□□□□□□□□□□□□■■■■■■■■■	1	Beijing	0
00216	2013	Kuala Lumpur	F/36	□□□□□□□□□□□□□□□□□□□□□□□□□□□□□□□□□□■■■■■■■■■	1	Beijing	0

00020	2011	Kelantan	M/74	■□□■■■■■■■■■■■■■■■■■■■■■■■■■□□□□■□■■□□□■■■■	11	EAI3-IND	1
00006	2010	Kelantan	M/70	■■□■■■■■■■■■■■■■■■■□□■■■■■■■□□□□■□■■■■■■■■■	19	EAI2-Manila	1
00022	2012	Kelantan	M/34	■■□■■■■■■■■■■■■■■■■□□■■■■■■■□□□□■□■■■■■■■■■	19	EAI2-Manila	1
00065	2012	Kelantan	F/47	■■□■■■■■■■■■■■■■■■■□□■■■■■■■□□□□■□■■■■■■■■■	19	EAI2-Manila	1
00072	2012	Kelantan	M/67	■■□■■■■■■■■■■■■■■■■□□■■■■■■■□□□□■□■■■■■■■■■	19	EAI2-Manila	1
00077	2012	Kelantan	F/18	■■□■■■■■■■■■■■■■■■■□□■■■■■■■□□□□■□■■■■■■■■■	19	EAI2-Manila	1
00130	2013	Kelantan	F/25	■■□■■■■■■■■■■■■■■■■□□■■■■■■■□□□□■□■■■■■■■■■	19	EAI2-Manila	1
00165	2011	Kelantan	M/54	■■□■■■■■■■■■■■■■■■■□□■■■■■■■□□□□■□■■■■■■■■■	19	EAI2-Manila	0
00208	2013	Kuala Lumpur	F/55	■■□■■■■■■■■■■■■■■■■□□■■■■■■■□□□□■□■■■■■■■■■	19	EAI2-Manila	0
00218	2013	Kuala Lumpur	M/38	■■□■■■■■■■■■■■■■■■■□□■■■■■■■□□□□■□■■■■■■■■■	19	EAI2-Manila	0
00215	2013	Kuala Lumpur	M/31	■■■□□□□■■■■■■■■■■■■■■■□□□□□□□□□□□□■■■■■■■■■	26	CAS1-Delhi	0
00031	2011	Kelantan	M/63	■■■■■■■■□□■■■■■■■■■■■■■■■■■■■■■■□□□□■■■■■■■	34	S	1
00143	2013	Kelantan	M/37	■■■■■■■■■■■■■■■■■■■■■■■■■■■■□□□□■□■■■■■□■■■	48	EAI1-SOM	1
00067	2011	Kelantan	F/70	■■■■■■■■■■■■■■■■■■■■■■■■■■■■■■□■□□□□■■■■■■■	50	H3	1
00118	2013	Kelantan	F/22	■■■■■■■■■■■■■■■■■■■■■■■■■■■■■■□■□□□□■■■■■■■	50	H3	1
00212	2013	Kuala Lumpur	M/60	■■■■■■■■■■■■■■■■■■■■■■■■■■■■■■□■□□□□■■■■■■■	50	H3	0
00004	2010	Kelantan	F/62	■■■■■■■■■■■■■■■■■■■■■■■■■■■■■■■■□□□□■■■□□□□	51	T1	1
00188	2013	Kuala Lumpur	M/63	■■■■■■■■■■■■■■■■■■■■■■■■■■■■■■■■□□□□■■■□□□□	51	T1	0
00206	2013	Kuala Lumpur	M/53	■■■■■■■■■■■■■■■■■■■■■■■■■■■■■■■■□□□□■■■■■■■	53	T1	0
00176	2011	Kelantan	M/46	■■■■■■■■■■■■■■■■■■■■■■■■■■■■■■■■□□■■■■■■■■■	54	Manu2	0
00005	2011	Kelantan	M/34	■■□■■■■□□□□□□□□□□□□□□□□□□■■■□□□□■□■■■■■■■■■	89	EAI2-nonthaburi	1
00023	2012	Kelantan	F/23	■■□■■■■□□□□□□□□□□□□□□□□□□■■■□□□□■□■■■■■■■■■	89	EAI2-nonthaburi	1
00082	2012	Kelantan	M/49	■■□■■■■□□□□□□□□□□□□□□□□□□■■■□□□□■□■■■■■■■■■	89	EAI2-nonthaburi	1
00090	2012	Kelantan	F/58	■■□■■■■□□□□□□□□□□□□□□□□□□■■■□□□□■□■■■■■■■■■	89	EAI2-nonthaburi	1
00116	2013	Kelantan	M/43	■■□■■■■□□□□□□□□□□□□□□□□□□■■■□□□□■□■■■■■■■■■	89	EAI2-nonthaburi	0
00068	2011	Kelantan	F/25	■□□■■■■■■■■■■■■■■■■■■■■■■■■■□□□□■□■■■■■■■■■	126	EAI5	3 (STR-R)
00009	2011	Kelantan	M/22	■■■■■■■■■■■■■■■■■■■■■■■■■■■■□□□□■□■■■■■■■■■	236	EAI5	0
00012	2010	Kelantan	F/65	■■■■■■■■■■■■■■■■■■■■■■■■■■■■□□□□■□■■■■■■■■■	236	EAI5	3 (STR-R)
00018	2012	Kelantan	M/25	■■■■■■■■■■■■■■■■■■■■■■■■■■■■□□□□■□■■■■■■■■■	236	EAI5	1
00047	2011	Kelantan	F/16	■■■■■■■■■■■■■■■■■■■■■■■■■■■■□□□□■□■■■■■■■■■	236	EAI5	1
00049	2011	Kelantan	M/39	■■■■■■■■■■■■■■■■■■■■■■■■■■■■□□□□■□■■■■■■■■■	236	EAI5	0
00081	2012	Kelantan	M/60	■■■■■■■■■■■■■■■■■■■■■■■■■■■■□□□□■□■■■■■■■■■	236	EAI5	1
00083	2012	Kelantan	M/32	■■■■■■■■■■■■■■■■■■■■■■■■■■■■□□□□■□■■■■■■■■■	236	EAI5	1
00153	2013	Kelantan	M/69	■■■■■■■■■■■■■■■■■■■■■■■■■■■■□□□□■□■■■■■■■■■	236	EAI5	0
00154	2013	Kelantan	F/76	■■■■■■■■■■■■■■■■■■■■■■■■■■■■□□□□■□■■■■■■■■■	236	EAI5	0
00155	2013	Kelantan	M/16	■■■■■■■■■■■■■■■■■■■■■■■■■■■■□□□□■□■■■■■■■■■	236	EAI5	1
00021	2012	Kelantan	F/23	□□□□□□□□□□□□□□□□□□□□□□□□□□□□□□□□□□□□□■■■■■■	250	Beijing	1
00207	2013	Kuala Lumpur	M/53	□□□□□□□□□□□□□□□□□□□□□□□□□□□□□□□□□□□□□■■■■■■	250	Beijing	0
00034	2010	Kelantan	M/36	■■■■■■■■■■■■■■■■■■■■■■■■■■■■□□□□■□■■■■□■■■■	256	EAI5	1
00064	2011	Kelantan	M/29	■■■■■■■■■■■■■■■■■■■■■■■■■■■■□□□□■□■■■■□■■■■	256	EAI5	1
00080	2012	Kelantan	M/68	■■■■■■■■■■■■■■■■■■■■■■■■■■■■□□□□■□■■■■□■■■■	256	EAI5	1
00100	2012	Kelantan	M/82	■■■■■■■■■■■■■■■■■■■■■■■■■■■■□□□□■□■■■■□■■■■	256	EAI5	1
00101	2012	Kelantan	M/69	■■■■■■■■■■■■■■■■■■■■■■■■■■■■□□□□■□■■■■□■■■■	256	EAI5	1
00115	2013	Kelantan	F/39	■■■■■■■■■■■■■■■■■■■■■■■■■■■■□□□□■□■■■■□■■■■	256	EAI5	1
00125	2013	Kelantan	F/57	■■■■■■■■■■■■■■■■■■■■■■■■■■■■□□□□■□■■■■□■■■■	256	EAI5	1
00140	2013	Kelantan	M/70	■■■■■■■■■■■■■■■■■■■■■■■■■■■■□□□□■□■■■■□■■■■	256	EAI5	1
00160	2009	Kelantan	F/54	■■■■■■■■■■■■■■■■■■■■■■■■■■■■□□□□■□■■■■□■■■■	256	EAI5	0
00175	2011	Kelantan	M/37	■■■■■■■■■■■■■■■■■■■■■■■■■■■■□□□□■□■■■■□■■■■	256	EAI5	0
00179	2011	Kelantan	F/28	■■■■■■■■■■■■■■■■■■■■■■■■■■■■□□□□■□■■■■□■■■■	256	EAI5	0
00184	2013	Kelantan	M/60	■■■■■■■■■■■■■■■■■■■■■■■■■■■■□□□□■□■■■■□■■■■	256	EAI5	0
00074	2011	Kelantan	M/82	□□□□□□□□□□□□□□□□□□□□□□□□□□□□□□□□□□■■□□■■■■■	260	Beijing	0
00124	2013	Kelantan	M/54	■□□■■■■■■■■■■■□■■■■■■■■■■■■■□□□□■□■■□□□■■■■	298	EAI3-IND	1
00132	2013	Kelantan	M/60	■□□■■■■□□□■■■■■■■■■■■■■■■■■■□□□□■□■■■■■■■■■	340	EAI5	1
00050	2012	Kelantan	F/82	■■■■■■■■■■■■□■■■■■■■■■■■■■■■□□□□■□■■■■■□■■■	349	EAI1-SOM	1
00038	2010	Kelantan	M/35	■■■■■■■■■■■■■■■■■■■■■■■■■■□□□□□□□□□□■■■■■■■	374	Unknown	1
00214	2013	Kuala Lumpur	F/49	■■■□□□□■■■■■■■■■■■■■■■□□□□□□□□□□□□■■■■■□■■■	429	CAS1-Delhi	0
00203	2013	Kuala Lumpur	F/48	■■□■■■■■■■■■■■■■■■■□□■■■■■■■□□□□■□■■■■■□□□■	483	EAI2-Manila	0
00162	2012	Kelantan	M/40	■■■■■■■■■■■■■■■■■■■■□■■■■■■■□■■■□□□□■■□■■■■	581	T1	0
00008	2011	Kelantan	F/61	■■■■■■■■■■■■■■■■■■■■■■□■■■■■□□□□■□■■■■■■■■■	591	EAI6-BGD1	**2 (MDR)**
00027	2011	Kelantan	M/64	■■■■■■■■■■■■■■■■■■■■■■□■■■■■□□□□■□■■■■■■■■■	591	EAI6-BGD1	0
00030	2011	Kelantan	M/47	■■■■■■■■■■■■■■■■■■■■■■□■■■■■□□□□■□■■■■■■■■■	591	EAI6-BGD1	1
00036	2011	Kelantan	F/55	■■■■■■■■■■■■■■■■■■■■■■□■■■■■□□□□■□■■■■■■■■■	591	EAI6-BGD1	1
00058	2011	Kelantan	M/52	■■■■■■■■■■■■■■■■■■■■■■□■■■■■□□□□■□■■■■■■■■■	591	EAI6-BGD1	1

00071	2010	Kelantan	M/82	■■■■■■■■■■■■■■■■■■■■■■□■■■■■□□□□■□■■■■■■■■■	591	EAI6-BGD1	0
00092	2012	Kelantan	M/73	■■■■■■■■■■■■■■■■■■■■■■□■■■■■□□□□■□■■■■■■■■■	591	EAI6-BGD1	1
00107	2012	Kelantan	M/51	■■■■■■■■■■■■■■■■■■■■■■□■■■■■□□□□■□■■■■■■■■■	591	EAI6-BGD1	1
00127	2013	Kelantan	F/68	■■■■■■■■■■■■■■■■■■■■■■□■■■■■□□□□■□■■■■■■■■■	591	EAI6-BGD1	1
00151	2013	Kelantan	M/38	■■■■■■■■■■■■■■■■■■■■■■□■■■■■□□□□■□■■■■■■■■■	591	EAI6-BGD1	1
00167	2011	Kelantan	M/23	■■■■■■■■■■■■■■■■■■■■■■□■■■■■□□□□■□■■■■■■■■■	591	EAI6-BGD1	0
00174	2011	Kelantan	M/64	■■■■■■■■■■■■■■■■■■■■■■□■■■■■□□□□■□■■■■■■■■■	591	EAI6-BGD1	0
00194	2013	Kuala Lumpur	M/33	■■■■■■■■■■■■■■■■■■■■■■□■■■■■□□□□■□■■■■■■■■■	591	EAI6-BGD1	0
00032	2011	Kelantan	M/28	■■■■■■■■■■■■■■■■■■■■■■□■■■■■■■■■■■■■■■■■■■■	623	Unknown	1
00192	2013	Kuala Lumpur	M/66	■□□■■■■■■■■■■■■■■■■■■■■■■■■■■■□■□□□□■■■■■■■	655	H3	0
00205	2013	Kuala Lumpur	F/35	■□□■■■■■■■■■■■■■■■■■■■■■■■■■■■□■□□□□■■■■■■■	655	H3	0
00003	2011	Kelantan	M/50	■■■■■■■■■■■■■■■■□■■■■■□□■■■■□□□□■□■■■■■■■■■	733	EAI6-BGD1	1
00002	2012	Kelantan	M/71	■■■■■■■■■■■■■■■■■■■■■■■■■■■■□□□□■□■■□□■□■■■	745	EAI1-SOM	3 (STR-R)
00007	2011	Kelantan	M/67	■■■■■■■■■■■■■■■■■■■■■■■■■■■■□□□□■□■■□□■□■■■	745	EAI1-SOM	1
00010	2012	Kelantan	M/39	■■■■■■■■■■■■■■■■■■■■■■■■■■■■□□□□■□■■□□■□■■■	745	EAI1-SOM	1
00025	2011	Kelantan	M/79	■■■■■■■■■■■■■■■■■■■■■■■■■■■■□□□□■□■■□□■□■■■	745	EAI1-SOM	1
00033	2011	Kelantan	F/21	■■■■■■■■■■■■■■■■■■■■■■■■■■■■□□□□■□■■□□■□■■■	745	EAI1-SOM	1
00042	2011	Kelantan	F/26	■■■■■■■■■■■■■■■■■■■■■■■■■■■■□□□□■□■■□□■□■■■	745	EAI1-SOM	1
00046	2011	Kelantan	M/89	■■■■■■■■■■■■■■■■■■■■■■■■■■■■□□□□■□■■□□■□■■■	745	EAI1-SOM	1
00048	2012	Kelantan	F/65	■■■■■■■■■■■■■■■■■■■■■■■■■■■■□□□□■□■■□□■□■■■	745	EAI1-SOM	1
00051	2011	Kelantan	M/73	■■■■■■■■■■■■■■■■■■■■■■■■■■■■□□□□■□■■□□■□■■■	745	EAI1-SOM	3 (RIF-R)
00053	0000	Kelantan	M/55	■■■■■■■■■■■■■■■■■■■■■■■■■■■■□□□□■□■■□□■□■■■	745	EAI1-SOM	1
00062	2010	Kelantan	F/67	■■■■■■■■■■■■■■■■■■■■■■■■■■■■□□□□■□■■□□■□■■■	745	EAI1-SOM	1
00066	2012	Kelantan	F/78	■■■■■■■■■■■■■■■■■■■■■■■■■■■■□□□□■□■■□□■□■■■	745	EAI1-SOM	1
00088	2012	Kelantan	F/32	■■■■■■■■■■■■■■■■■■■■■■■■■■■■□□□□■□■■□□■□■■■	745	EAI1-SOM	1
00095	2012	Kelantan	F/79	■■■■■■■■■■■■■■■■■■■■■■■■■■■■□□□□■□■■□□■□■■■	745	EAI1-SOM	1
00097	2012	Kelantan	M/60	■■■■■■■■■■■■■■■■■■■■■■■■■■■■□□□□■□■■□□■□■■■	745	EAI1-SOM	1
00099	2012	Kelantan	M/78	■■■■■■■■■■■■■■■■■■■■■■■■■■■■□□□□■□■■□□■□■■■	745	EAI1-SOM	1
00102	2012	Kelantan	M/62	■■■■■■■■■■■■■■■■■■■■■■■■■■■■□□□□■□■■□□■□■■■	745	EAI1-SOM	1
00111	2012	Kelantan	M/55	■■■■■■■■■■■■■■■■■■■■■■■■■■■■□□□□■□■■□□■□■■■	745	EAI1-SOM	1
00117	2013	Kelantan	F/52	■■■■■■■■■■■■■■■■■■■■■■■■■■■■□□□□■□■■□□■□■■■	745	EAI1-SOM	1
00119	2013	Kelantan	F/66	■■■■■■■■■■■■■■■■■■■■■■■■■■■■□□□□■□■■□□■□■■■	745	EAI1-SOM	1
00122	2013	Kelantan	M/71	■■■■■■■■■■■■■■■■■■■■■■■■■■■■□□□□■□■■□□■□■■■	745	EAI1-SOM	1
00123	2013	Kelantan	F/52	■■■■■■■■■■■■■■■■■■■■■■■■■■■■□□□□■□■■□□■□■■■	745	EAI1-SOM	1
00131	2013	Kelantan	F/45	■■■■■■■■■■■■■■■■■■■■■■■■■■■■□□□□■□■■□□■□■■■	745	EAI1-SOM	1
00135	2013	Kelantan	M/50	■■■■■■■■■■■■■■■■■■■■■■■■■■■■□□□□■□■■□□■□■■■	745	EAI1-SOM	1
00137	2013	Kelantan	F/21	■■■■■■■■■■■■■■■■■■■■■■■■■■■■□□□□■□■■□□■□■■■	745	EAI1-SOM	1
00142	2013	Kelantan	M/27	■■■■■■■■■■■■■■■■■■■■■■■■■■■■□□□□■□■■□□■□■■■	745	EAI1-SOM	1
00145	2013	Kelantan	M/49	■■■■■■■■■■■■■■■■■■■■■■■■■■■■□□□□■□■■□□■□■■■	745	EAI1-SOM	1
00163	2011	Kelantan	M/28	■■■■■■■■■■■■■■■■■■■■■■■■■■■■□□□□■□■■□□■□■■■	745	EAI1-SOM	0
00169	2011	Kelantan	M/82	■■■■■■■■■■■■■■■■■■■■■■■■■■■■□□□□■□■■□□■□■■■	745	EAI1-SOM	0
00173	2011	Kelantan	F/65	■■■■■■■■■■■■■■■■■■■■■■■■■■■■□□□□■□■■□□■□■■■	745	EAI1-SOM	0
00180	2011	Kelantan	M/56	■■■■■■■■■■■■■■■■■■■■■■■■■■■■□□□□■□■■□□■□■■■	745	EAI1-SOM	0
00181	2011	Kelantan	M/43	■■■■■■■■■■■■■■■■■■■■■■■■■■■■□□□□■□■■□□■□■■■	745	EAI1-SOM	0
00191	2013	Kuala Lumpur	M/6	■■■■■■■■■■■■■■■■■■■■■■■■■■■■□□□□■□■■□□■□■■■	745	EAI1-SOM	0
00069	2011	Kelantan	F/23	■■■■■■■□■■■■■■■■■■■■■■■■■■■■□□□□■□■■■■■■■■■	939	EAI5	3 (STR-R)
00091	2012	Kelantan	M/63	■■■■■■■■■■■■■■■■■■■■■■■■■□□□□□□□□□□□□□□■■■■	944	Unknown	1
00172	2011	Kelantan	F/62	■■■■■■■■■■■■■■■■■■■■■■□□□□□□□□□■□□□□■■■■■■■	946	H	0
00141	2013	Kelantan	M/76	■■■■■■■□□□□■■■■■■■■■■■■■■■■■□□□□■□■■■■■■■■■	951	EAI5	1
00078	2012	Kelantan	M/56	■■■■■■■■■■■■■■■■■■■■■■■■■■■■□□□□■□■■□□□□■■■	962	EAI5	3 (STR-R)
00161	2010	Kelantan	F/72	■■■■■■■■■■■■■■■■■■■■■■■■■■■■□□□□■□■■□■■□■■■	1183	EAI1-SOM	0
00045	2011	Kelantan	M/48	□□□□□□□□□□□□□□□□■□□□□□□□□□□□□□□□□□■■■■■■■■■	1184	Unknown	1
00156	2013	Kelantan	M/40	■■■■■■■■■□■■■■■■■■■■■■■■■■■■■■□■□□□□■■■■■■■	1243	H3	0
00189	2013	Kuala Lumpur	M/24	■■■■■■■■■■■■■■□□□□□□□■□■■■■■■■■□□□□■■■■■■■	1268	T5	0
00152	2013	Kelantan	F/70	■■■■■■■■■■□■■■■■■■■■■■■■■■■■□□□□■□■■■■■■■■■	1372	EAI5	1
00204	2013	Kuala Lumpur	F/42	■□□■■■■□□□■■■■■■■■■■■■■□■■■■□□□□■□■■■■■■■■■	1395	EAI5	0
00219	2013	Kuala Lumpur	F/40	■■□■■■■■■■■■■■□■■■■□□■■■■■■■□□□□■□■■■■■■■■■	1490	EAI2-Manila	0
00054	2011	Kelantan	M/43	■■■■■■■■■■■■■■■■■■■■□■■■■■□■■■■■□□□□■■■■■■■	1494	T1	1
00041	2011	Kelantan	M/65	■■□■□■■■■■■■■■■■■■■□□■■■■■■■□□□□■□■■■■■□□□■	1512	EAI2-Manila	3 (RIF-R)
00202	2013	Kuala Lumpur	M/55	□□□□□□□□□□□□□□□□□□□□□□□□□□□□□□□□□□■■■■□■■□■	1651	Beijing	0
00211	2013	Kuala Lumpur	M/67	□□□□□□□□□□□□□□□□□□□□□□□□□□□□□□□□□□■■■■□■■□■	1651	Beijing	0
00013	2011	Kelantan	M/29	■■■■□■■■■■■■□■■■■■■■□□□□■■■■■■■■□□□□■■■■■■■	2383	LAM5	3 (STR-R)
00139	2013	Kelantan	F/33	■■□■■■■□□□□□□□■■■■■■□□□□■■■■■■■■□□□□■■■■■■■	2428	LAM3	1

00076	2012	Kelantan	M/58	□□□□□□□□□□□□□□□□□□□□□□□□□□□□□□□□□□■■■■■■■■□	2610	Beijing	1
00177	2011	Kelantan	F/59	□□□□□□□□□□□□□□□□□□□□□□□□□□□□□□□□□□■■■■■■■■□	2610	Beijing	0
00190	2013	Kuala Lumpur	F/42	■■■■■■■■■■■■■■□■■■■■■■■■■■■■■■■■□□□□■■■□□□□	3143	T1	0
00029	2011	Kelantan	M/45	■■■□■■■■■■■■■■■■■■■■■■□■■■■■□□□□■□■■■■■■■■■	3484	EAI6-BGD1	3 (RIF-R)
00014	2011	Kelantan	M/35	■□□■■■■■■■■■■■■■■■■■■■■■■■■■□□□□■□■■□□□□□□■	3949	EAI5	1
00039	2011	Kelantan	M/57	■■■■■■■■■■■■■■■■■■■■■■■■■■■■□□□□■□□□□□□□■■■	3993*	EAI5	1
00060	2011	Kelantan	F/23	■■■■■■■■■■■■□■■■■■■■■■■■■□■■□□□□■□■■■■■□■■■	3994*	EAI1-SOM	1
00109	2012	Kelantan	M/67	■■■■■■■■■■■■□■■■■■■■■■■■■□■■□□□□■□■■■■■□■■■	3994*	EAI1-SOM	1
00070	2011	Kelantan	M/61	■■■■■■■■□■■■■■■■■■■■■■■■■■■□□□□■□■■■■■■■■■	3995*	EAI5	1
00121	2013	Kelantan	F/61	■■■■■■■■□■■■■■■■■■■■■■■■■■■□□□□■□■■■■■■■■■	3995*	EAI5	1
00098	2012	Kelantan	M/21	■■■■■■■■■■■■■■■■■■■■□□■■■□□□□□□□□□□□□□□■■■■	3996*	Unknown	1
00108	2012	Kelantan	F/58	■■■■■■■■■■■■■■■■■■■■□□■■■□□□□□□□□□□□□□□■■■■	3996*	Unknown	1
00149	2013	Kelantan	M/50	■■■■■■■■■■■■■■■■■■■■■■■■■■■■□□□□■□■■□□■□□□■	3997*	EAI5	1
00171	2011	Kelantan	M/60	■■■■■■■■■■■■■■■■■■■■■■■■■■■■□□□□■□■■□□■□□□■	3997*	EAI5	0
00164	2011	Kelantan	M/55	■■■■□□□□□□□□■■■■■■■■■■□■■■■■□□□□■□■■■■■■■■■	3998*	EAI6-BGD1	0
00168	2011	Kelantan	F/66	■■■■□□□□□□□□■■■■■■■■■■□■■■■■□□□□■□■■■■■■■■■	3998*	EAI6-BGD1	0
00019	2012	Kelantan	M/59	■□□■■■■□□□□□■■■■■■■■■■■■■■■■□□□□■□■■□□□■■■■	Or01	EAI3-IND	1
00024	2011	Kelantan	M/53	■■■■■■■■■■■■■■■■■■■■■■□■■■■■□□□□■□■■■□■■■■■	Or02	EAI6-BGD1	1
00028	2011	Kelantan	M/49	■□□■■■■■■■■■■■■■□□□■■■■■■■□■□□□□■□■■□□□■■■■	Or03	EAI3-IND	1
00044	2011	Kelantan	M/38	□□□□□□□□□□□□□□□□■□□□□□□□□□□□□□□□□■■□■■■■■■	Or04	Unknown	1
00055	2011	Kelantan	M/89	■■■■■■■■■■■■■■■■■■■■■■■■■■■□□□□□■□■■■■□■■■■	Or05	EAI5	1
00059	2011	Kelantan	F/60	■■■■■■■■■■■■■■■■■■□■■■■■■■■■□□□□■□■■□□■□■■■	Or06	EAI1-SOM	1
00073	2012	Kelantan	M/40	■■■■■■■■■■■□□■■■■■■■■■■■■■■■□□□□■□■■□□■□■■■	Or07	EAI1-SOM	1
00085	2012	Kelantan	F/51	■■■■■■■□■■■■■■■■■□■■■■■■■■■■□□□□■□■■■■■□■■■	Or08	EAI1-SOM	1
00086	2012	Kelantan	F/68	■■■■■■■□□□□□□□□□□□□□□□□□□□□■□□□□■□■■□□■□■■■	Or09	EAI1-SOM	1
00114	2013	Kelantan	F/22	■■■■■■■□■■■■■■■■■■■■■■■■■■■■□□□□■□■■□□■□■■■	Or10	EAI1-SOM	1
00128	2013	Kelantan	F/48	■■■□■■■□■■■■□■■■■■■■■■■■■■■■□□□□■□■■■■■■■■■	Or11	EAI5	1
00133	2013	Kelantan	M/45	■■■■■■■■■■■■■■■■■■□■■■■■■■■■□□□□■□■■■■■□■■■	Or12	EAI1-SOM	1
00136	2013	Kelantan	F/13	■■■■■■■■■■■■■■■■■■■■■□■■■■■■□□□□■□■■□□■□■■■	Or13	EAI1-SOM	1
00146	2013	Kelantan	M/69	■■■■■■■□■■■■■■■■■■■■■■■■■■■■□□□□■□■■■■■□■■■	Or14	EAI1-SOM	1
00158	2009	Kelantan	M/14	■■□□■■□■□□■□■□□□■■■■■■■■■■□■■■■□■■■■■■□□□□□	Or15	BOV	0
00159	2009	Kelantan	M/30	■■■■■■■■■■■■■■■■■□■■■■■■■■■■□□□□■□■■■■□□■■■	Or16	EAI5	0
00166	2011	Kelantan	F/37	■■■■□■■■■■■■■■■■■■■■■■□□□□□□□□□■□□□□■■■■■■■	Or17	H3	0
00178	2011	Kelantan	F/41	■■■□■■■■■■■■□□□■■■□■■■■■■■■■■■■■□□□□■■■■■■■	Or18	T4	0
00185	2013	Kuala Lumpur	M/25	■■■■■■■■■■■■□■□■■■■■□□□□□□□□□□□■□□□□■■□■■□■	Or19	H3	0
00196	2013	Kuala Lumpur	M/38	■■■■□■■■■■■■□■□■■■■■■■■■■■■■■■■■□□□□■■■□■■■	Or20	T	0
00217	2013	Kuala Lumpur	F/65	■■■■■■■■■■■□□□■■■■■■■■■□■■■■■■■■■■■■■■■■■■■	Or21	Unknown	0
00220	2013	Kuala Lumpur	M/46	■■■■■■■■■■■■■■■■□■■■■■■■■■■■□□□□■□■■■■■□■■■	Or22	EAI5	0

[a] Note that for orphan spoligotype patterns (n=22 strains marked as Or01-Or22, highlighted in blue), lineages were assigned manually by Expert-based interpretations using SITVITWEB rules.

[b] Note that SITs followed by an asterisk (n=6 patterns containing 11 strains, highlighted in yellow) indicate "newly created shared-types" due to 2 or more strains belonging to an identical new pattern within this study or after a match with an orphan in the database; SIT designations followed by number of strains: 3993* this study n=1, MYS n=1; 3994* this study n=2, MYS n=1; 3995* this study n=2; 3996* this study n=2; 3997* this study n=2; 3998* this study n=2.

[c] Drug resistance code; “0” for unknown; “1” for strain susceptible to all first-line drugs; “2” for MDR-TB (combined resistance to INH-RIF); “3” for any other resistances (followed by the name of the drugs: STR, Streptomycin; INH, Isoniazid; RIF, Rifampin; ETB, Ethambutol; PZA, Pyrazinamide).
